# Fecal microbiota composition is a better predictor of recurrent *Clostridioides difficile* infection than clinical factors in a prospective, multicentre cohort study

**DOI:** 10.1186/s12879-024-09506-7

**Published:** 2024-07-10

**Authors:** Tessel M. van Rossen, Yvette H. van Beurden, Johannes A. Bogaards, Andries E. Budding, Chris J.J. Mulder, Christina M.J.E. Vandenbroucke-Grauls

**Affiliations:** 1 Department of Medical Microbiology & Infection Control, Amsterdam UMC, Vrije Universiteit Amsterdam, Amsterdam, The Netherlands; 2 Amsterdam Institute for Infection and Immunity, Amsterdam, The Netherlands; 3 Department of Gastroenterology & Hepatology, Amsterdam UMC, Vrije Universiteit Amsterdam, Amsterdam, The Netherlands; 4 Amsterdam Gastroenterology Endocrinology Metabolism Institute, Amsterdam, The Netherlands; 5 Department of Epidemiology and Data Science, Amsterdam UMC, Vrije Universiteit Amsterdam, Amsterdam, The Netherlands; 6 Amsterdam Public Health, Amsterdam, The Netherlands; 7 Inbiome, Amsterdam, The Netherlands; 8 Department of Clinical Epidemiology, Aarhus University, Aarhus, Denmark

## Abstract

**Introduction:**

*Clostridioides difficile* infection (CDI) is the most common cause of antibiotic-associated diarrhoea. Fidaxomicin and fecal microbiota transplantation (FMT) are effective, but expensive therapies to treat recurrent CDI (reCDI). Our objective was to develop a prediction model for reCDI based on the gut microbiota composition and clinical characteristics, to identify patients who could benefit from early treatment with fidaxomicin or FMT.

**Methods:**

Multicentre, prospective, observational study in adult patients diagnosed with a primary episode of CDI. Fecal samples and clinical data were collected prior to, and after 5 days of CDI treatment. Follow-up duration was 8 weeks. Microbiota composition was analysed by IS-pro, a bacterial profiling technique based on phylum- and species-specific differences in the 16–23 S interspace regions of ribosomal DNA. Bayesian additive regression trees (BART) and adaptive group-regularized logistic ridge regression (AGRR) were used to construct prediction models for reCDI.

**Results:**

209 patients were included, of which 25% developed reCDI. Variables related to microbiota composition provided better prediction of reCDI and were preferentially selected over clinical factors in joint prediction models. Bacteroidetes abundance and diversity after start of CDI treatment, and the increase in Proteobacteria diversity relative to baseline, were the most robust predictors of reCDI. The sensitivity and specificity of a BART model including these factors were 95% and 78%, but these dropped to 67% and 62% in out-of-sample prediction.

**Conclusion:**

Early microbiota response to CDI treatment is a better predictor of reCDI than clinical prognostic factors, but not yet sufficient enough to predict reCDI in daily practice.

**Supplementary Information:**

The online version contains supplementary material available at 10.1186/s12879-024-09506-7.

## Introduction

*Clostridioides difficile* infection (CDI) is the most common cause of hospital-associated diarrhoea in the developed world. Despite adequate treatment, 25–30% of patients develop recurrent CDI (reCDI) [1, 2]. This leads to severe morbidity, mortality, and high costs. In the recently updated European and North American treatment guidelines for CDI, fidaxomicin is preferred over vancomycin for the treatment of an initial episode of CDI because of lower recurrence risk (reCDI rate fidaxomicin 12.7–19.5% vs. vancomycin 25.3–26.9%) [3, 4]. As fidaxomicin is expensive, vancomycin or metronidazole remain the standard treatment for economic reasons in many regions, but these drugs are associated with substantial recurrence risk. Fecal microbiota transplantation (FMT) is advised for patients with CDI recurrence(s) [5].

Identifying patients at risk of reCDI is challenging as many factors are associated with reCDI. Several clinical prognostic factors for reCDI have been identified and used to develop prediction models [6–15]. Nevertheless, external validation of these tools has shown disappointing results; to the best of our knowledge, no clinical prediction model is sufficiently robust for use in daily practice [16]. Since the gut microbiota plays an essential role in CDI, identification of patients at risk of reCDI on the basis of gut microbial features may provide a better alternative.

The objective of this study was to develop a prediction model for reCDI based on the gut microbiota composition, combined with *C. difficile* ribotype (RT, since certain ribotypes such as NAP1/027 strain are associated with a higher risk of reCDI), and clinical characteristics during the first episode of CDI [17]. Such a combined prediction model could be used to stratify patients with regard to their recurrence risk and help clinicians to identify patients who could benefit from FMT or fidaxomicin for their primary CDI.

## Materials and methods

### Study population

This prospective, observational study was carried out in a university hospital and five large community hospitals between March 2018 and December 2021. All patients (≥ 18 years) with an initial episode of CDI treated with metronidazole, vancomycin or fidaxomicin were eligible for inclusion. CDI was defined as the presence of diarrhoea (≥ 3 unformed stools per 24 h) in combination with a positive *C. difficile* toxin EIA (enzyme immunoassay) and/or positive *C. difficile* toxin gene NAAT (nucleic acid amplification test). Patients with CDI in the preceding three months, microbiologically proven infectious enterocolitis (other than CDI) in the last month, or ileostomy were excluded. The study endpoint was reCDI, including primary non-responders and patients with recurrence after initial treatment response. Primary non-response was defined as persistent diarrhoea during and for at least two days after completion of CDI treatment, in combination with a positive *C. difficile* toxin EIA and/or toxin gene NAAT. Recurrence after initial response was defined as recurrent diarrhoea within 8 weeks from the day of CDI diagnosis, after resolution of the initial symptoms for at least two days and after completion of CDI treatment, in combination with a positive *C. difficile* toxin EIA and/or toxin gene NAAT. A sensitivity analysis excluding primary non-responders is provided in the Supplementary. This study was approved by the medical ethical research committee of Amsterdam UMC (approval number 2015.299). Written informed consent was obtained from all participants.

### Data collection

Data on demographics, medical history, disease severity and medication use were collected prospectively by (telephone) interviews and verified and completed with electronic patient healthcare records by a small group of trained researchers. Patients were contacted by telephone on day 4, 10, 14, 28 and 56 after CDI diagnosis to evaluate disease course and potential reCDI occurrence. Participants were asked to contact the study coordinator if they developed diarrhoea between the scheduled time points. Total follow-up duration was 56 days (8 weeks).

### Sample collection

Aliquots of samples sent to the microbiology laboratory for routine testing for CDI (obtained prior to CDI treatment) were stored at -20 °C for *C. difficile* surveillance purposes. Samples of patients who provided informed consent were included in this study. An additional fecal sample was collected on day 4, 5 or 6 after initiation of CDI treatment. For patients admitted to the hospital, these samples were sent to the microbiology laboratory and stored at -20 °C. When patients were at home, this second fecal sample was collected at home in a sterile container and stored in the patient’s own freezer [18]. All samples were transported to the research laboratory on dry ice and stored at -20 °C until further handling.

### Laboratory analysis

#### DNA extraction

DNA was extracted from 200 to 400 mg of feces using the chemagic DNA stool kit according to the manufacturer’s instructions, and using a chemagic 360 machine (PerkinElmer chemagen, Baesweiler, Germany, protocol chemagic DNA Stool 360 VD201021).

#### Microbiota analysis

Microbiota analysis was performed with the Molecular Culture Microbiota kit (Inbiome, Amsterdam, the Netherlands), according to manufacturer’s instructions. This assay is based on the IS-pro technique (Molecular Culture, Inbiome, Amsterdam, the Netherlands), a bacterial profiling method, based on bacterial species-specific differences in the length and number of the 16–23 S IS-regions of the ribosomal DNA, with taxonomic classification by phylum-specific fluorescent labelling of PCR primers (Supplementary methods) [19]. The fragment length (in nucleotides) represents a bacterial species and is considered an operational taxonomic unit (OTU), while the intensity (in relative fluorescent units, RFU) determines the abundance. Potentially clinically relevant fragments were linked to specific bacterial species via the IS-pro species database (Inbiome, Amsterdam, the Netherlands), containing data on IS-fragment lengths of previously cultured or sequenced species. The IS-pro technique has been proven to be an efficient and informative method to study (gut) microbial communities for clinical applications, and results are comparable to those obtained by 16 S sequencing [20–25].

#### *C. difficile* ribotyping

*C. difficile* ribotyping was performed directly on fecal DNA as described previously [26]. The mastermix was kindly provided by Inbiome (Amsterdam, the Netherlands). Ribotype was assessed as predictor for reCDI as binary value: hypervirulent strain (ribotype 027 or 078) vs. other strain.

### Statistical analysis

First, we assessed the clinical and microbiota characteristics of the study population. Secondly, we investigated possible associations between these clinical and microbial factors. Finally, we developed several prediction models for reCDI with different combinations of clinical and/or microbial factors, to identify the model with the best performance.

#### Analysis of clinical data

Differences in clinical factors, at baseline or at day 5 of treatment, with regard to reCDI at day 56 of follow-up, were assessed by standard tests. For semi-continuous variables, we employed Student’s t-test or the Mann–Whitney U test, depending on distribution of data. For categorical variables, either the Chi-square test or Fisher’s exact test was used, depending on expected cell frequencies.

We applied Bayesian additive regression trees (BART) to investigate the joint performance of all 72 clinical factors on which we had sufficient data (listed in the Supplementary) for the prediction of reCDI [27, 28]. BART is well-suited for studying non-linear relationships and has been shown to perform particularly well when the number of candidate predictors is of the same order as the number of samples (p ≈ n). Out-of-sample performance of BART was assessed by means of 10-fold cross-validation.

#### Analysis of microbiota data

Microbiota data were either analysed at the species level (abundance of each IS-fragment) or features summarized in terms of phylum-specific microbial abundances and Shannon diversities, calculated from the phylum-specific profile of IS-pro fragment length distribution. Prediction models based on microbiota summary measures were constructed with BART, and out-of-sample performance was assessed by 10-fold cross-validation. Prediction models based on microbiota profiles (abundance of all IS-fragments) were constructed with adaptive group-regularized logistic ridge regression (AGRR). In contrast to BART, this method can only identify linear associations between reCDI risk and each predictor variable, but it enables more efficient estimation and predictor selection when the number of features (e.g. over 3700 IS-fragments) far exceeds the number of samples [29, 30]. Furthermore, it allows the use of co-data (e.g. phylum information) and co-variates (e.g. clinical characteristics) to improve predictive performance. Predictive performance was assessed by Receiver Operating Characteristic (ROC) Curves and Area Under these Curves (AUC) based on out-of-sample predictions obtained from 10-fold cross-validation. We assessed whether addition of microbiota summary measures could improve the prediction of reCDI based on individual IS-fragments only, by adding them as fixed, i.e. non-penalized, covariates to the model, or as flexible covariates subject to regularized selection.

#### Joint prediction by clinical and microbiome data

We investigated whether addition of microbiota summary measures to BART models improved prediction. We also assessed whether the addition of clinical factors could improve the predictive performance of models based on IS-fragments. To this end, we performed a two-stage prediction procedure, by embedding selection of clinical factors via BART within the cross-validation loops of AGRR, with or without addition of microbiota summary measures added as fixed covariates to the model.

Finally, we considered a full joint analysis on all clinical factors, IS-fragments and microbiota summary measures, collected either at baseline or at day 5 of treatment, into one predictor. Different penalization of distinct types of data (clinical predictors, individual IS-fragments, microbiota summary measures) was achieved through specification of appropriate classes of co-data.

All statistical analyses were performed with R using the packages ‘vegan’, ‘GRridge’ and ‘bartMachine’, built under R version 4.1.1. More information on statistical analysis is provided in the Supplement.

## Results

### Clinical characteristics

A total of 209 patients were included in the study. Fifty-two patients (25%) developed reCDI: of these, 41 patients (79%) developed a recurrence after initial treatment response and 11 patients (21%) were primary non-responders. The reCDI group contained more alcohol users, immunocompromised patients, IBD patients, and severe CDI cases than the non-reCDI group (Table 1). Patients who developed reCDI were significantly less often hospitalized on the day of primary CDI diagnosis, and had used antibiotics significantly less often in the 10 days preceding primary CDI diagnosis, as compared to patients who did not develop reCDI. Lastly, patients who developed reCDI were more often treated with vancomycin, and less often with metronidazole. Only three patients were treated with fidaxomicin; none developed reCDI. Presence of an hypervirulent *C. difficile* strain (ribotype 027 or 078) was similar in both groups.

**Table 1:** Clinical characteristics

Clinical characteristics	Total population *n* (%)	No reCDI *n* (%)	reCDI *n* (%)	
Number of patients	209 (100.0)	157 (75.1)	52 (24.9)	
Age, mean [SD]	66.2 [16.8]	66.4 [16.9]	65.5 [17.0]	
Female gender	110 (52.6)	82 (52.2)	28 (53.8)	
Alcohol users‡	98 (46.9)	71 (45.2)	28 (53.8)	
Immunocompromised	85 (40.7)	59 (37.6)	26 (50.0)	
IBD	17 (8.1)	11 (7.0)	6 (11.5)	
Type of hospital of enrolment				
*Academic*	45 (21.5)	33 (21.0)	12 (23.1)	
*General*	164 (78.5)	124 (79.0)	40 (76.9)	
Hospitalized on day of CDI diagnosis	180 (86.1)	142 (90.4)	38 (73.1)	*
Location of CDI onset/association				
*HCF-onset, HCF-associated*	104 (49.8)	83 (52.9)	21 (40.4)	
*Community-onset, HCF-associated*	44 (21.1)	33 (21.0)	11 (21.2)	
*Community-onset, community-associated*	50 (23.9)	33 (21.0)	17 (32.7)	
*Indeterminate disease*	11 (5.3)	8 (5.1)	3 (5.8)	
Hypervirulent strain (ribotype 027 or 078)	12 (6.6) of *n* = 183†	9 (6.5) of *n* = 138†	3 (6.7) of *n* = 45†	
Severe CDI	76 (36.4)	54 (34.4)	22 (42.3)	
Antibiotic for CDI				
*Metronidazole*	115 (55.0)	91 (58.0)	24 (46.2)	
*Vancomycin*	89 (42.6)	62 (39.5)	27 (51.9)	
*Fidaxomicin*	3 (1.4)	3 (1.9)	0	
*Unknown*	2 (1.0)	1 (0.6)	1 (1.9)	
Antibiotic use within 10 days before start of primary CDI treatment	116 (55.5)	97 (61.8)	19 (36.5)	*
Antibiotic use within 3 months (and excl. 10 days) before start of primary CDI treatment	140 (67.0)	103 (65.6)	37 (71.2)	
Proton pump inhibitor‡	119 (56.9)	91 (58.0)	28 (53.8)	

HCF: healthcare facility; ‡within the 3 months before primary CDI diagnosis; †Percentages based on available stool samples: at baseline stool samples were obtained from 183/209 patients; 45/52 reCDI patients and 138/157 non-reCDI patients; * *p* *< 0.05*

### Microbiota characteristics

**Fig. 1:**
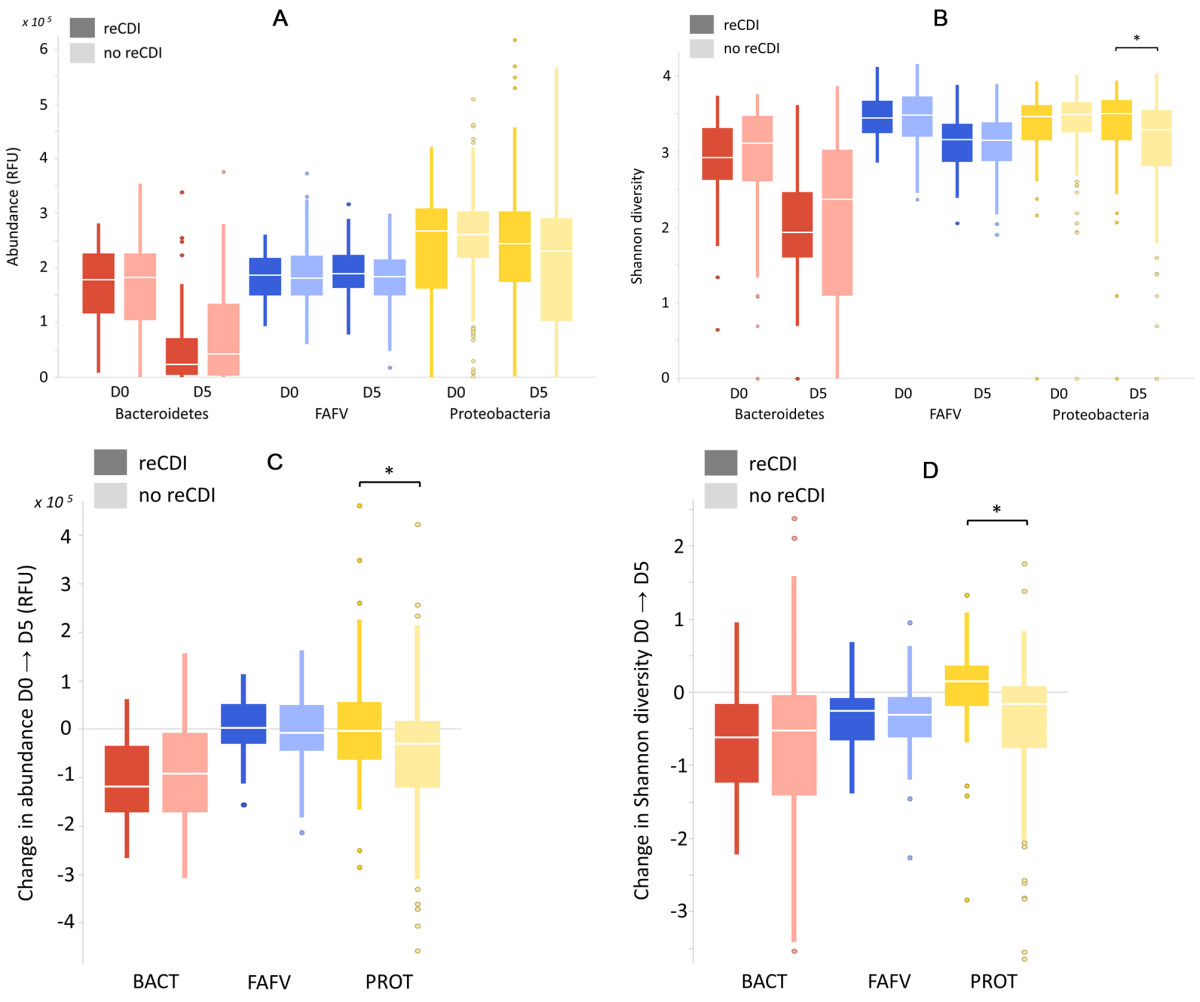
Boxplots (depicting median, interquartile ranges and outliers) of microbial abundance **(A)**, diversity **(B)** and changes in abundance **(C)** and diversity **(D)** between D0-D5 in patients with treatment failure (dark bars) and patients with treatment success (light bars). Statistically significant differences between reCDI and non-reCDI patients are indicated (*). *D0: at baseline, before start of CDI treatment; D5: 5 days after start of CDI treatment; FAFV: Firmicutes, Actinobacteria, Fusobacteria, Verrucomicrobia; BACT: Bacteroidetes; PROT: Proteobacteria*

Microbial abundance and diversity of samples collected prior to CDI treatment (day 0, D0) and after 5 days of CDI treatment (D5), and respective changes between D0 and D5, were determined per patient and are visualized in Fig. 1. In all patients, Bacteroidetes abundance and diversity were extremely reduced after initiation of CDI treatment (Fig. 1A and B). On D5, patients with reCDI had a significantly higher Proteobacteria diversity than patients without reCDI (Fig. 1B). In addition, they had lower Bacteroidetes abundance and diversity, although these differences were not statistically significant. With respect to changes in microbiota composition between D0 and D5, in non-reCDI patients the Proteobacteria abundance and diversity decreased, while in reCDI patients Proteobacteria abundance stayed stable, and the diversity even increased (Fig. 1C and D). *C. difficile* abundance was similar in reCDI and non-reCDI patients. For details, see Table S1.

### Association between clinical factors and microbial abundance or diversity

Clinical factors significantly associated with microbial abundance or diversity on D0 or D5 are listed in Table 2. Almost all types of antibiotics used in the 3 months before primary CDI diagnosis were associated with a lower Bacteroidetes abundance or diversity (compared to patients who had not used these antibiotics). The majority of the significant associations between clinical factors and microbiota on D0 were no longer present on D5 of CDI treatment, whereas only few associations appeared on D5 of CDI treatment (listed in the Supplementary). The type of CDI antibiotic had a large effect on microbiota composition on D5: patients who were treated with vancomycin had a higher Bacteroidetes abundance and diversity than patients who were treated with metronidazole. A detailed overview of results can be found in Tables S2-S4.

**Table 2:** Selection of clinical factors associated with microbial abundance/diversity at baseline or on day 5 of CDI treatment

Clinical factor	Microbiota composition on D0						Microbiota composition on D5					
	Abundance†			Diversity¶			Abundance†			Diversity¶		
	BACT	FAFV	PROT	BACT	FAFV	PROT	BACT	FAFV	PROT	BACT	FAFV	PROT
Age ≥ 65 years				▲		▲						
Female gender	▼			▼								
Tobacco use ‡			▼			▼						
Hospitalization on D0					▼				▼			
Enteral feeding ‡					▼							
IBD		▲			▲			▲	▲			▲
Immunocompromised ‡		▲		▼								
Loose stool type on D5			▼									▼
*Prior antibiotic use*: ‡												
Carbapenem	▼					▼						▼
Cotrimoxazole	▼	▲		▼								
Aminoglycoside				▼							▼	
Against anaerobes #				▼	▼							
Metronidazole	▼	▲		▼		▼	▼			▼		
Vancomycin/teicoplanine		▲										
Antibiotic continuation D5					▼			▼	▼		▲	▼
CDI antibiotic vancomycin*							▲			▲		

Only variables are listed for which at least one comparison (absolute abundance or Shannon diversity) yielded *p* < 0.05 by the Mann-Whitney U test. Clinical factors that were associated with microbial abundance or diversity on D5 but not on D0 are not shown, except for type of CDI antibiotic, since CDI treatment was initiated at D0. Upward/downward arrows indicate the difference in Phylum-specific abundance/diversity (higher/lower respectively) in patients in which the specific clinical factor is present, compared to patients in which it is absent. *IBD: Inflammatory Bowel Disease; FAFV: Firmicutes, Actinobacteria, Fusobacteria, Verrucomicrobia; BACT: Bacteroidetes; PROT: Proteobacteria;* † *Absolute abundances and log abundance ratios;* ¶ *Shannon diversity on log-transformed abundances (adding one to handle zeroes) of all true peaks in each channel*; ‡within 3 months before start of CDI treatment; # penicillins, 1st generation cephalosporines, carbapenems, clindamycin, macrolides, tetracyclines and/or metronidazole; *vs. metronidazole

### Association between clinical factors and bacterial species

On D0, the strongest associations between clinical factors and microbiota composition at bacterial species level were observed for prior use of cotrimoxazole (AUC 0.76) and hospitalization on the day of CDI diagnosis (AUC 0.72). In line with the previous observation that hospitalized patients had a lower FAFV diversity than non-hospitalized patients (Table 2), hospitalization was associated with a decrease of mainly FAFV species, such as *Akkermansia muciniphila*, *Faecalibacterium prausnitzii*, *Ruminococcus gnavus*, and several Clostridium and Eubacterium species. In contrast, *Fusobacterium nucleatum* and *Bacteroides vulgatus* were increased in hospitalized patients. Also, the use of several antibiotics in the 3 months preceding CDI diagnosis was strongly associated with microbiota composition prior to CDI treatment (AUC ≥ 0.70, see Table S5).

On day 5 of CDI treatment, compared to D0, several associations between clinical variables and bacterial species were reduced or not present anymore. However, hospitalization remained strongly associated with microbiota composition (AUC 0.76). The strongest association on D5 was between type of CDI treatment (vancomycin vs. metronidazole) and microbiota composition (AUC 0.88). This was mainly based on lower abundances of several FAFV species in vancomycin users, compared to metronidazole users (Table S5).

### Prediction models for reCDI

#### Prediction model based on clinical factors

**Fig. 2:**
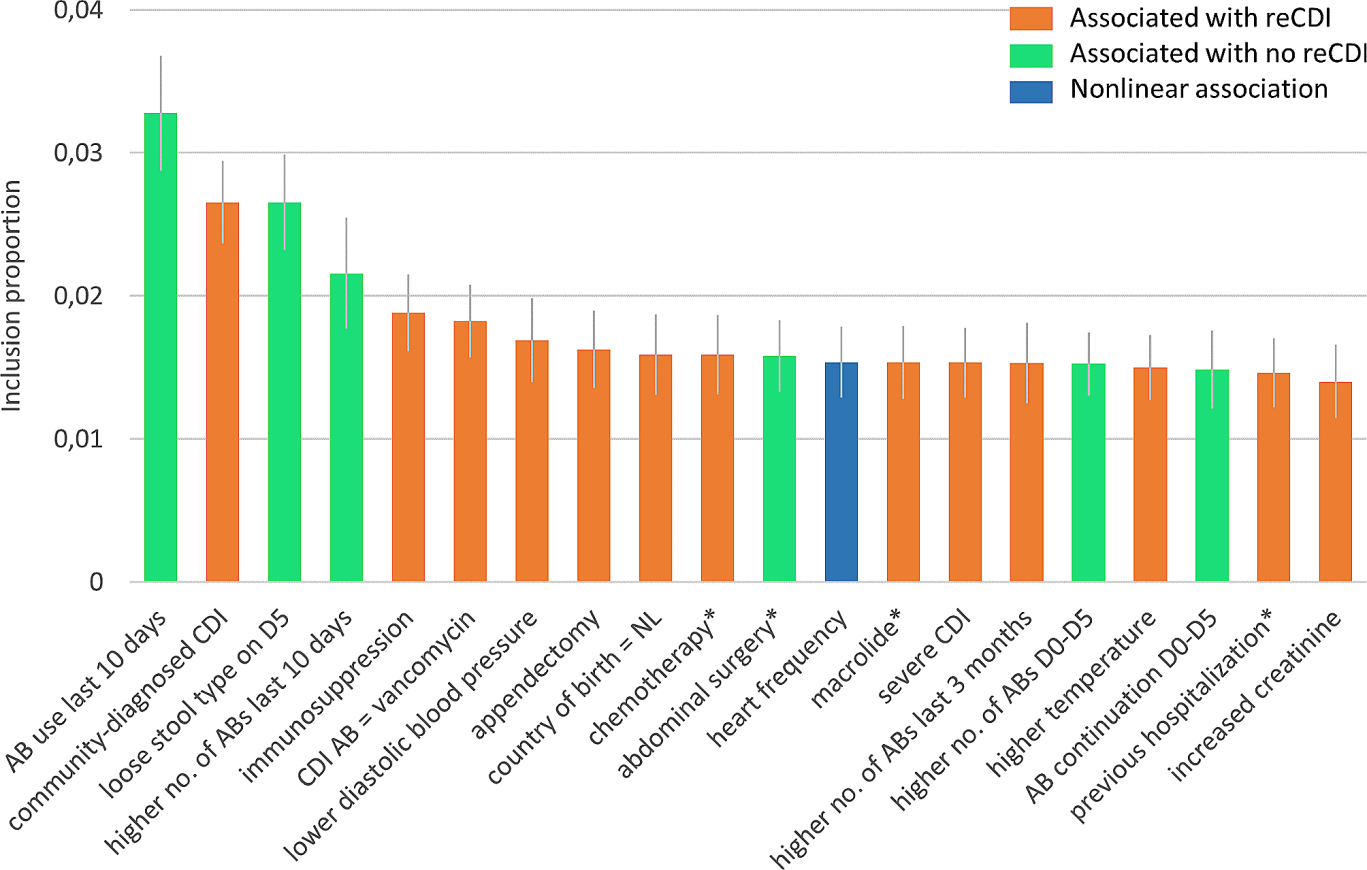
The twenty most important clinical factors for reCDI prediction. Inclusion proportion refers to the proportion of decision nodes in which the clinical factor was included; the higher the inclusion proportion, the more important the clinical factor is for predicting reCDI (vs. no reCDI). The blue bar indicates that the association between heart frequency and reCDI is not linear, having an optimum at intermediate values (see partial effect plots in Figure S1. *within 3 months before start of CDI treatment

To predict reCDI based on clinical characteristics, we used BART. Seventy-two clinical factors with sufficient data were included (Supplementary text 1). The twenty most important clinical factors for the prediction of reCDI are shown in Fig. 2. The prediction model on all 209 patients yielded a sensitivity and specificity of both 56% after 10-fold cross validation, indicating poor generalizability of the model to patients whose characteristics were not used for model building.

### Prediction models based on microbial factors

**Fig. 3:**
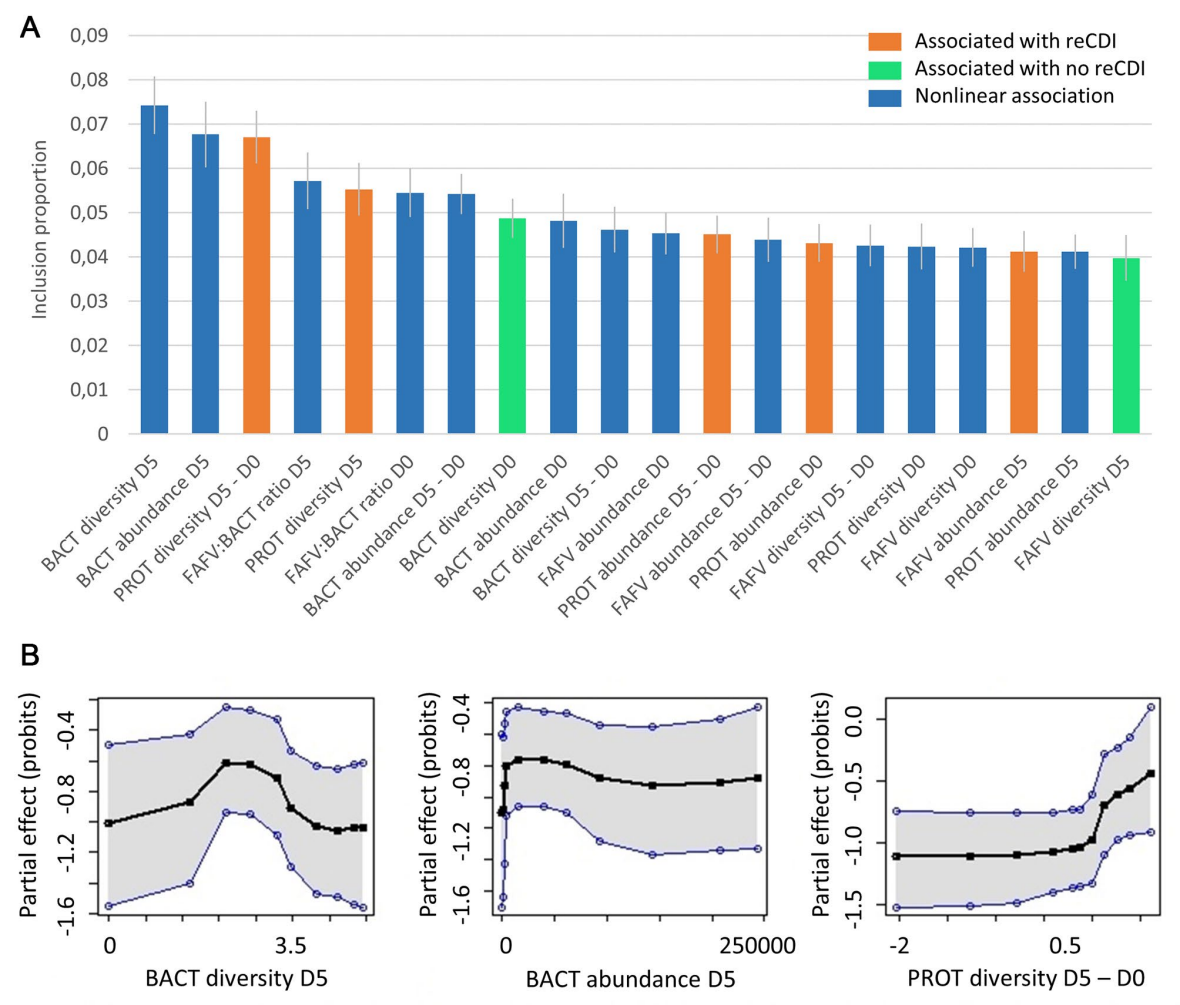
**A** The twenty most important microbial abundance/diversity factors for reCDI prediction. Inclusion proportion refers to the proportion of decision nodes in which the clinical factor is included; the higher the inclusion proportion, the more important the factor is for predicting reCDI. The blue bars indicate nonlinear associations, having an optimum at intermediate values (see Fig. 3B and Figure S2). **B** Partial effect plots of the three most important microbial factors for prediction of reCDI, provided by BART. These plots show the association between a predictor (in this case, a specific microbial abundance/diversity) and the outcome (reCDI risk) for any given value of the predictor. Therefore, in case of non-linear associations this model provides more accurate predictions than for example logistic regression, which can only identify linear associations (described by regression coefficients). The higher the partial effect (Y-axis), the higher the chance of reCDI. For all partial effect plots, see Figure S2

Next, a (BART) prediction model for reCDI was developed based on microbial abundance and diversity. Bacteroidetes diversity and abundance on D5, and the difference in Proteobacteria diversity between D0 and D5, were the strongest predictors of reCDI. As shown in Fig. 3, the associations between many microbial factors and treatment failure were not linear. The model based on microbial abundance and diversity had a better performance than the model based on clinical factors, and yielded a sensitivity of 67% and a specificity of 62% in out-of-sample prediction.

We then developed a prediction model for reCDI based on all IS-fragments using AGRR. The performance of this model was assessed by determining AUC values. Patients with and without reCDI could clearly be distinguished based on bacterial species on D0 or D5 of their primary CDI episode, thus before reCDI had occurred (AUC 1.0 and 0.97, respectively, Table 3 and S6). However, after cross-validation these AUCs decreased to 0.46 and 0.42, indicating poor generalizability to the complete study population due to overfitting on the patients used for model building. Next, we assessed whether a model based on differences in bacterial species between D0 and D5, and models based on a combination of bacterial species and microbial abundance/diversity increased predictive performance, but this was not the case (AUCs 0.41–0.56, Table S6).

**Table 3:** Prediction models for reCDI based on combinations of clinical factors, bacterial species, and/or microbial abundance/diversity (before CDI treatment, D0). Colours indicate whether the clinical/microbial factor is associated with an increased (orange) or decreased (green) reCDI risk. First the number and Phylum (FAFV/BACT/PROT) of IS-fragments associated with reCDI are listed, and then the bacterial species that were matched to these fragments via the IS-pro species database. Prediction models on D5 had a similar or worse performance and are shown in Table S6

Prediction model based on:	AUC	AUC after cross-validation	Specification of most distinctive factors
Bacterial species on D0	1.	0.46	9 FAFV, 7 BACT, 2 PROT, 1 FAFV, 6 BACT: *Bacteroides fragilis, Bacteroides thetaiotaomicron, Clostridium perfringens, Clostridium sporogenes, Desulfovibrio vulgaris, Dialister spp., Micrococcus luteus, Parabacteroides merdae, Ruminococcus gnavus Bacteroides uniformis, Faecalibacterium prausnitzii, Prevotella fusca*
Clinical factors and bacterial species on D0	1.	0.49	Antibiotics last 10 days, no. of antibiotics last 10 days, 8 FAFV, 6 BACT, 3 PROT, 3 FAFV, 3 BACT: *Bacteroides fragilis, Bacteroides thetaiotaomicron, Clostridium perfringens, Coprococcus eutactus, Desulfovibrio vulgaris, Dialister spp., Micrococcus luteus, Parabacteroides merdae, Ruminococcus gnavus, Sutterella wadsworthensis, Bacteroides uniformis, Faecalibacterium prausnitzii, Prevotella fusca, Streptococcus equi*
Preselection of clinical factors‡ and flexible selection of bacterial species on D0	1.	0.67	CDI antibiotic = vancomycin, stool type, antibiotics last 10 days, no. of antibiotics last 10 days, 1 FAFV, 3 BACT, 4 FAFV, 3 BACT, 2 PROT: *Bacteroides thetaiotaomicron, Desulfovibrio vulgaris, Fusobacterium spp. ISF179, Parabacteroides merdae (2x), Faecalibacterium prausnitzii, Prevotella fusca, Proteobacteria ISP1124.*
Preselection of clinical factors‡, flexible selection of bacterial species and fixed$ inclusion of microbial abundance/diversity on D0	0.91	0.62	Immunocompromised, CDI antibiotic = vancomycin, stool type, antibiotics last 10 days, no. of antibiotics last 10 days, FAFV + BACT + PROT diversities and abundances, 2 FAFV, 1 BACT, 6 PROT, 1 BACT: *Desulfovibro vulgaris, Fusobacterium spp. ISF179, Parabacteroides merdae*

‡ *Pre-selection of patient characteristics or microbiota summary measures by Bayesian additive regression trees (BART) within cross-validation; factors selected in > 50% of cross-validation loops are listed, $ Fixed summary measures are not penalized, whereas flexible summary measures are penalized (see Supplementary methods)*

### Prediction models based on combinations of clinical and microbial factors

**Fig. 4:**
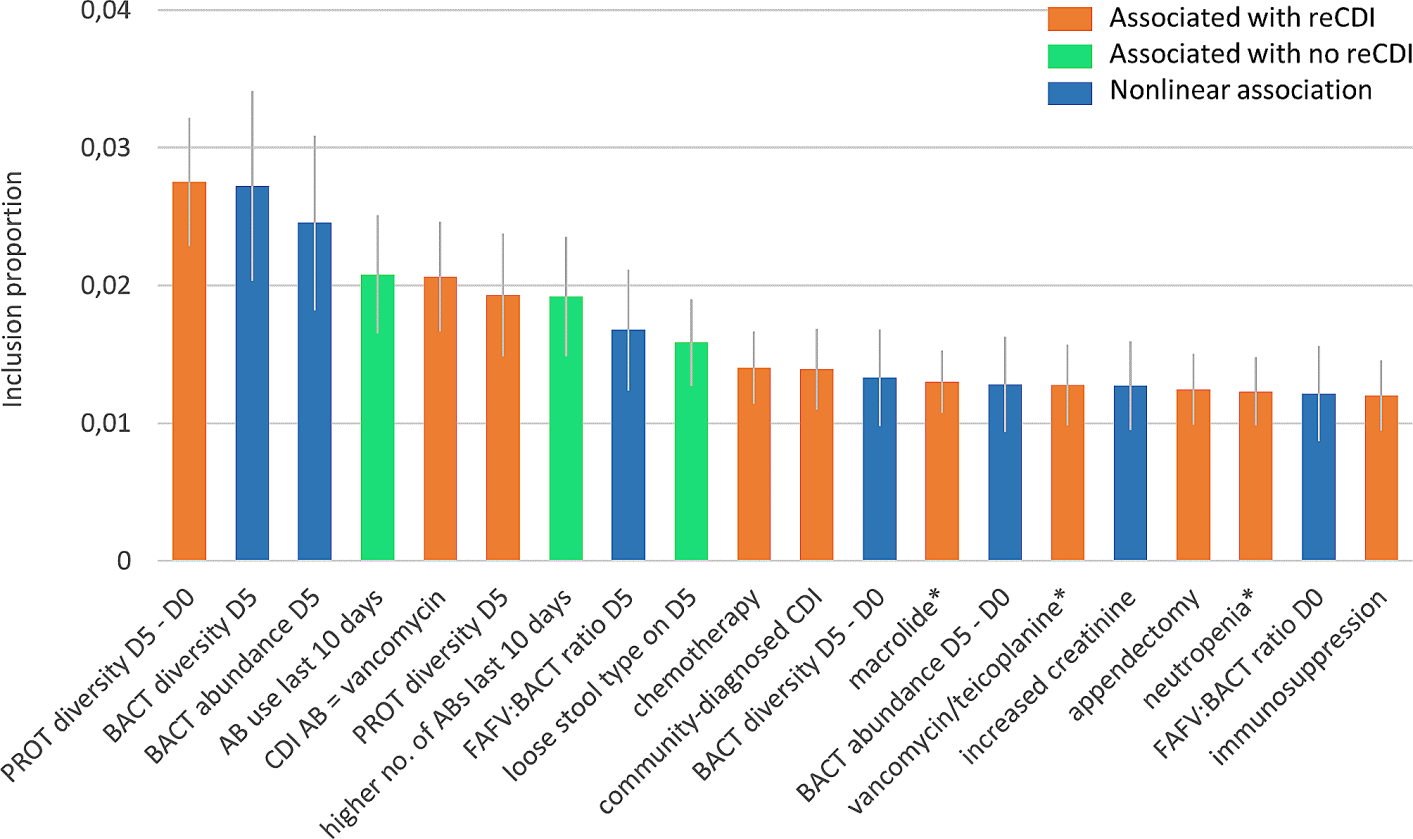
The twenty most important clinical and microbial abundance/diversity factors for reCDI prediction. Inclusion proportion refers to the proportion of decision nodes in which the clinical factor is included; the higher the inclusion proportion, the more important the factor is for predicting reCDI. The blue bars indicate nonlinear associations, having an optimum at intermediate values

Clinical factors and microbial abundance/diversity were combined in one prediction model with BART. In this combined model, the three strongest predictors of reCDI were the same as in the model with only microbial abundance/diversity: the difference in Proteobacteria diversity between D0 and D5, and Bacteroidetes diversity and abundance on D5 (Fig. 4). The accuracy of this model was better than the model based on clinical factors, but similar to the model based on microbiota abundance/diversity only. However, also the performance of this combined model decreased after cross-validation, and the model containing only microbial factors retained the highest predictive performance (Table 4).

**Table 4:** Prediction of reCDI by clinical factors and/or microbial abundance/diversity at baseline or D5 of CDI treatment

	Before cross-validation		After cross-validation	
Prediction based on:	Sensitivity	Specificity	Sensitivity	Specificity
Clinical factors	85%	71%	56%	56%
Microbial abundance/diversity	95%	78%	67%	62%
Clinical factors and microbial abundancy/diversity	95%	77%	60%	59%

Predictions are obtained by Bayesian additive regression trees (BART) with choice of the hyperparameters based on cross-validation

Next, clinical factors and bacterial species were combined in one prediction model with AGRR, because of the high number of possible predictors. Including all clinical factors and bacterial species on D0 yielded an AUC of 0.49 after cross-validation (Table 3). We attempted to reduce overfitting and to improve generalizability by decreasing the number of variables in the model to the most predictive clinical factors, as identified by BART within each cross-validation loop of AGRR. With an optimum number of three clinical factors in combination with bacterial species (Table S7, Figure S3), this improved the AUC to 0.67. Increased abundances of *Faecalibacterium prausnitzii* and *Prevotella fusca* on D0 were associated with lower reCDI risk, while several other Clostridium (a.o. *Clostridium perfringens*) and Bacteroides species, and *Fusobacterium spp.* were more prevalent in patients who developed reCDI.

**Fig. 5:**
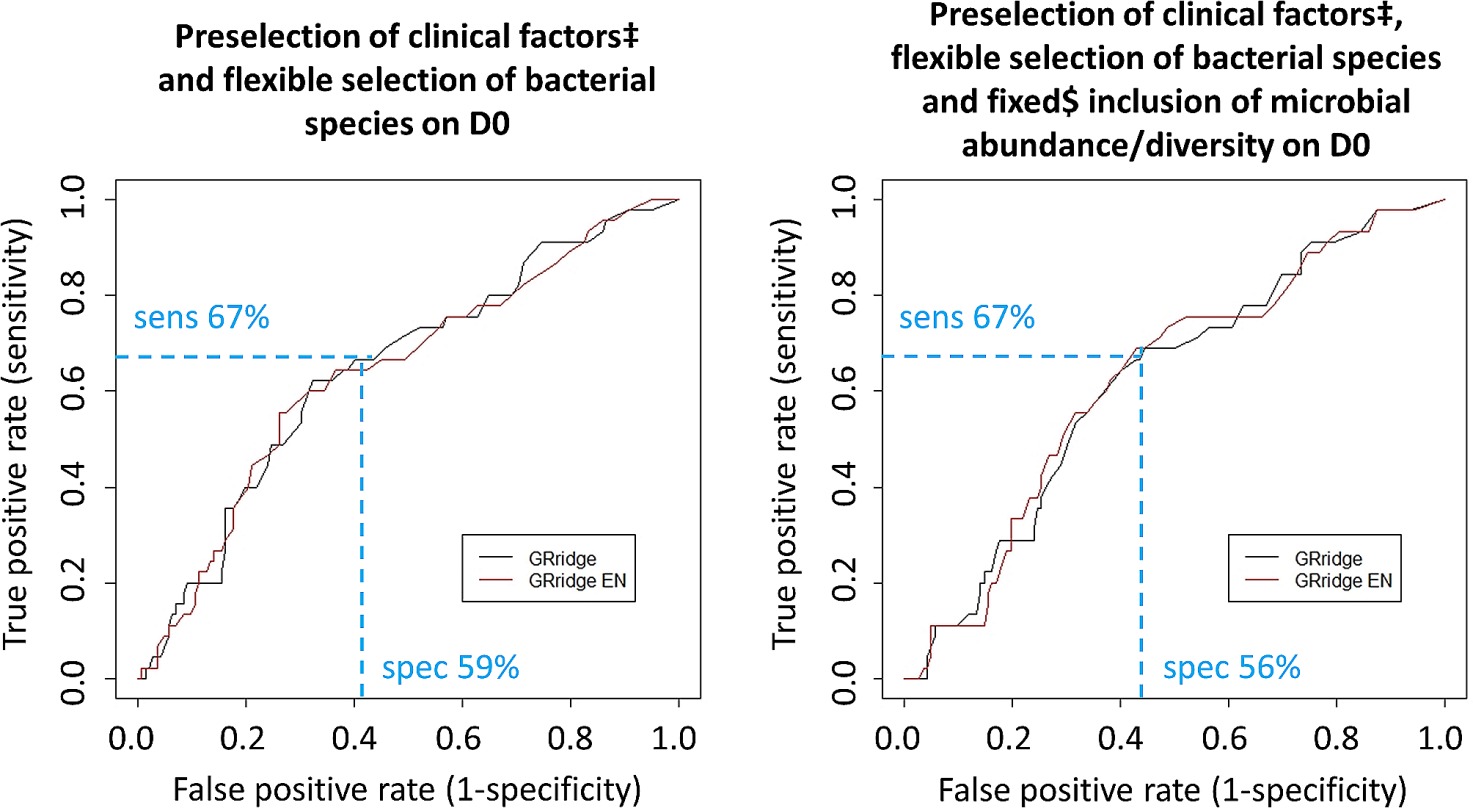
Receiver operating characteristic (ROC) curves of the two best performing AGRR models for the prediction of reCDI. For each model, the performance based on all factors (black) and based on a panel of the 25 most important factors via elastic-net (EN) feature selection (red) are shown. In blue, the sensitivity and corresponding specificity of the BART model based on microbial abundance/diversity is indicated

Finally, AGRR models were constructed with combinations of clinical factors, microbial abundance/diversity, and bacterial species on D0 or D5. The models including a preselection of three clinical factors (as described previously) and fixed microbial/abundance variables (see *Methods*) on D0 or D5 yielded AUCs after cross-validation of 0.62 and 0.63 (Table 3 and S6). The ROC curves of these two models are shown in Fig. 5. To compare the performance of these models to the BART model with the highest accuracy (i.e., based on microbial abundance/diversity only), we indicated the sensitivity of the BART model in these ROC curves. In the AGRR model based on a preselection of clinical factors and (not selected) bacterial species, this corresponded to a specificity of 59%, and in the model based on a preselection of clinical factors, bacterial species and microbial abundance/diversity, this corresponded to a specificity of 56%: slightly lower to the specificity of 62% of the model based on microbial abundance/diversity only. Additionally, we performed several sensitivity analyses, including an analysis excluding primary non-responders, but this did not lead to significantly better prediction accuracies (see Supplementary text 2). Lastly, we assessed possible interactions between bacterial phyla, and found that co-occurrence of Bacteroidetes abundance and Proteobacteria diversity within regression trees was low, indicating that these were largely independent predictors of reCDI (Supplementary text 3 and Figure S4).

## Discussion

In this study we found that microbiota composition was a better predictor of reCDI than clinical factors in a cohort of patients with a primary episode of CDI. Bacteroidetes abundance and diversity, and the difference in Proteobacteria diversity before and after start of CDI treatment, were the strongest predictors of reCDI. However, the sensitivity of 67% and specificity of 62% suggests that prediction tools based on clinical and/or microbial factors are not (yet) appropriate for prediction of reCDI in daily practice.

We also investigated possible associations between clinical and microbial factors. We found that the microbiota composition on D0 (before CDI treatment) was affected by many clinical factors such as age, gender, smoking, hospitalization, enteral feeding, IBD, immunosuppression, stool type and antibiotic use. Furthermore, we observed that the CDI treatment with either metronidazole or vancomycin had a large effect on the microbiota composition on day 5 of CDI treatment. The many interactions between host factors and microbiota composition highlight the complexity of predicting reCDI in a very heterogeneous population with respect to comorbidity and medication use.

In previous studies, several clinical prognostic factors for reCDI have been identified and multiple prediction models have been developed [6–15]. Nevertheless, probably due to the generally small effect of the identified predictors and low quality of studies, the performance of such models in external cohorts is disappointing [16]. This is in concordance with our findings that prediction of reCDI based on clinical characteristics seemed promising, but that the predictive value dropped to a sensitivity and specificity of both 56% after cross validation. This indicates the low predictive value of clinical factors, and the poor generalizability of prediction tools for reCDI based on clinical factors only.

The association between a disturbed intestinal microbiota and (re)CDI has been well-established [31–34]. However, most of these findings are derived from cross-sectional studies; prospective studies leading to a concrete prediction model for reCDI using microbiota composition are scarce [35–38]. Khanna et al. developed a risk score for reCDI based on a panel of most discriminating OTUs, which differentiated well between patients with and without reCDI (sensitivity 75%, specificity 69%, *n* = 88) [36]. In agreement with their findings, we found that a higher abundance of *Faecalibacterium prausnitzii* in pre-treatment samples was associated with less reCDI, while reCDI was associated with an increase in *Lachnospiraceae*, *Coprococcus*, *Parabacteroides*, *Ruminococcus gnavus*, and several *Clostridium* species, amongst which *Clostridium perfringens*. In concordance with another study (*n* = 31), in which a random forest model was developed based on bacterial species, we found that addition of clinical factors did not improve the predictive performance [37].

This study has several strengths. To the best of our knowledge, our work represents the largest study on microbiota-based prediction of reCDI. Due to the prospective design, we were able to collect data on more than seventy clinical factors, and obtained fecal samples both before and during primary CDI treatment. Furthermore, the microbiota assay we used (IS-pro/Molecular Culture) allows the assessment of absolute bacterial abundances, as opposed to relative abundances provided by next-generation sequencing. Absolute quantification is a prerequisite when using bacterial abundances over time and across patients in prediction models. Furthermore, the IS-pro technique has been proven to be an efficient and informative method to study (gut) microbial communities for clinical applications, and results are comparable to those obtained by 16 S sequencing, as previously shown in this journal [20–25]. Lastly, we applied statistical methods for high-dimensional data that are able to capture non-linear relationships with clinical outcome (BART) and incorporate hierarchical labelling of predictor variables (AGRR). Both methods rely on internal cross-validation for optimization of regularization parameters to deal with the large number of candidate predictors.

Despite the use of these techniques, overfitting could not be avoided, as shown by the decrease in performance of the various models in out-of-sample prediction. This is likely caused by the heterogeneity of our study population; compared to the population of Khanna et al., our patients were on average 13.5 years older, hence the number and variation of comorbidities and medications was possibly higher [36]. Another limitation of our study might be that primary non-responders and patients with recurrence after initial treatment response were combined in one primary endpoint (reCDI). However, a sensitivity analysis excluding primary non-responders did not improve prediction accuracy.

The observation that phylum-specific microbial abundance and diversity were better predictors of reCDI than individual bacterial species, might suggest that different clinical factors can induce similar changes in microbiota composition at the phylum level, which leads to the best discrimination between reCDI- and non-reCDI patients in this heterogeneous population. Apparently, these microbiota changes could not be narrowed down to the species level, possibly due to the large number of species compared to the number of patients. Another possible explanation is that one clinical factor might induce a certain functional change which is carried out by different bacterial species in different patients; these complex predictors and interactions may be detected by using a much larger sample size or functional assays such as metabolomics. Additionally, a model based on individual bacterial species is much more prone to overfitting and is less generalizable than a model based on microbiota summary measures. The predictive performance of a model based on bacterial species could be improved by adding a preselection of clinical factors, but the accuracy of such a complex model was similar to the relatively simple model based on summary measures only.

Our findings that a lower Bacteroidetes diversity and abundance, and an increase of Proteobacteria diversity were associated with the development of reCDI, are in agreement with previous studies on reCDI [37, 39]. This is in line with that Bacteroidetes are generally considered the most important members of a healthy core microbiota, while Proteobacteria are associated with microbiota dysbiosis and disease [40].

A seemingly surprising observation was that hospitalization on day of CDI diagnosis, and (any) antibiotic use in the 10 preceding days, was associated with a decreased risk of reCDI. One could expect that hospitalized patients with recent antibiotic exposure would have a more disturbed microbiota and therefore would be more prone to develop reCDI. However, it is crucial to realize that these factors are not compared between patients with and without CDI, but between patients who do or do not develop reCDI after an initial CDI episode. This introduces ‘index event bias’, which arises in studies that select patients based on the occurrence of an index event and evaluate recurrence, and can lead to ‘negative’ or even paradoxical findings with regard to variables known to be associated with the index event [41]. Another explanation for the association between recent antibiotic use and reCDI might be that patients with recent antibiotic use have a clear inciting factor for CDI and might therefore be more prone for successful CDI treatment, whereas patients without an evident trigger for CDI, might have a more definitive disturbed microbiota composition and are therefore more prone to treatment failure and reCDI.

In future studies, the prediction of reCDI might be improved by including larger sample sizes, which allows for stratification based on clinical and microbiological characteristics, and adjustment for index event bias [42]. The most efficient method to achieve this is by the construction of large, prospective CDI cohorts. This would allow for sharing and (re)using data and samples by scientists from different fields of expertise, saving time and money. Furthermore, promising microbial and host factors such as metabolomics, bile acids and immunologic markers should be further explored [38, 43, 44]. The limitation of such markers, in contrast to clinical factors and IS-pro-obtained microbiota data, is that they usually require special expertise and equipment and are therefore not easy to implement in daily clinical practice.

In conclusion, in our study population, microbiota composition was a better predictor of reCDI than clinical characteristics. We were not able to design a generalizable predictive model for reCDI, but identified important predictive factors (Bacteroidetes diversity and abundance, and the increase in Proteobacteria diversity after CDI treatment) that were also identified in previous studies. At present, clinicians should realize that each patient, regardless of clinical factors, might be at risk of reCDI.

### Electronic supplementary material

Below is the link to the electronic supplementary material.

Supplementary Material 1

## Data Availability

The datasets generated and/or analysed during the current study are available in the Figshare repository, 10.6084/m9.figshare.23503623.v1.
