# Supplementary material for: Fecal microbiota composition is a better predictor of recurrent Clostridioides difficile infection than clinical factors in a prospective, multicentre cohort study
Source: BMC Infect Dis. 2024 Jul 10;24:687. doi: 10.1186/s12879-024-09506-7 (PMC11238444; doi:10.1186/s12879-024-09506-7)

Supplementary

**Supplementary methods**

*IS pro analysis*

The IS-pro technique is a bacterial profiling method based on bacterial species-specific differences in the length and number of the 16S-23S IS-regions of the ribosomal DNA, with taxonomic classification by phylum-specific fluorescent labelling of PCR primers (15). The IS-pro assay combines two separate PCR reactions: the FIRBAC reaction, in which the bacterial phyla Firmicutes, Actinobacteria, Fusobacteria, Verrucomicrobia (FAFV) and Bacteriodetes are amplified, and the PROTEO reaction, in which Proteobacteria and the internal control are amplified. DNA fragments were separated by capillary gel electrophoresis using a ABI Prism 3500XL Genetic Analyzer (Applied Biosystems, Foster City, CA), analysed with fragment analyser version 7.0 (inbiome, Amsterdam, the Netherlands) and visualized with the Spotfire software package (TIBCO, Palo Alto, CA, USA). The color of each DNA fragment (‘peak’ within a microbiota profile) corresponds to a specific phylum. The fragment length (in nucleotides) represents a bacterial species and is considered an operational taxonomic unit (OTU), while the intensity (in relative fluorescent units, RFU) determines the abundance. Potentially clinically relevant fragments were linked to specific bacterial species via the IS-pro species database (inbiome, Amsterdam, the Netherlands), containing data on IS-fragment lengths of previously cultured or sequenced species.

*Bayesian Additive Regression Trees*

For use in prediction models we only considered those clinical variables with less than 10% missing values and at least 5% occurrence of the rarest category in case of binary variables. Variables thus selected were enriched by replacing missing values via generalized regression imputation. The resulting list of clinical variables with sufficient information is listed in Supplementary text 1. Thus, we used 72 clinical variables to predict 52 out of 209 events of reCDI.

To investigate the predictive performance of clinical variables with regard to reCDI, we used Bayesian Additive Regression Trees (BART) with probit link function. BART is a flexible, semi-parametric statistical machine learner to approximate unknown functional relationships and interactions between patient characteristics and response probabilities (20, 21). BART has been shown to perform particularly well as compared to other methods (e.g. random forests or gradient boosting) when the number of candidate predictors is in the order of the number of informative samples, as is the case in our application.

BART does not rely on strong functional and distributional assumptions and provides model-free importance rankings and interaction detection of candidate variables. To prevent overfitting, BART models are penalized by prior specifications. We used specifications as recommended by Chipman et al. [2010] and let BART determine hyperparameters via internal cross-validation. These relate to the number and branching depth of the regression trees to be built, as well as the number of candidate predictors to select from at each node. Out-of-sample performance of BART was assessed by means of 10-fold cross-validation. We used an empirical threshold for classification (based on the observed reCDI occurrence of approx. 25%) to translate the continuous risk score provided by BART into a binary prediction.

*Analysis of microbiome data*

Gut microbiome data as generated by IS-pro were analysed in two principal ways: by assessing associations with and predictive value of microbiota summary measures; and by assessing associations with and predictive value of specific fragment length abundance. The first analysis takes an ecological approach, whereas the second analyses searches for individual bacterial genera or species that might be of interest.

For the analysis on summary measures we calculated phylum-specific abundance and diversity from phyla defined by IS-pro: FAFV, Bacteroidetes, and Proteobacteria. Abundance was calculated as the summed abundance, as indicated by relative fluorescence units, of all true peaks within each phylum-specific channel of IS-pro. We also calculated the ratio of FAFV to Bacteroidetes abundance. Diversity was calculated from log-transformed abundances (adding one to handle zeroes) of all true peaks in each channel, using the Shannon diversity index function.

To assess the association of clinical outcome with specific microbial elements as determined by IS-pro, either at baseline of at day 5 of treatment, we first constructed a heatmap based on the 50 most discriminative fragments, as determined by pairwise t-testing on log-transformed relative fluorescence units. Pairwise associations were subsequently visually investigated by means of Manhattan plots, and putative associations were individually assessed by the Mann-Whitney U test with false discovery rate control by Benjamini-Hochberg correction.

*Adaptive group-regularized logistic ridge regression*

To assess the predictive value of IS-pro with regard to clinical outcome, we performed adaptive group-regularized logistic ridge regression and post hoc group-weighted elastic net feature selection as described before (22, 23). This classifier enables estimation and predictor selection when the number of features (in this case, bacterial features) far exceeds the number of samples. Furthermore, it allows for the structural use of co-data to improve predictive performance. Two types of auxiliary data were separately provided to the model for group regularization of the included fragments: (a) information on bacterial phylum: FAFV, Bacteroidetes, or Proteobacteria; and (b) information on average abundance and standard deviation of each IS-pro fragment in the analysis. A regression model was built with log-transformed IS-pro data collected either at baseline, at day 5 of treatment, or of the relative difference between timepoints. The 25 most discriminative fragments were selected by post hoc group-weighted elastic net feature selection, and out-of-sample performance of the model was evaluated by 10-fold cross-validation. Performance was visualized by receiver operating characteristic (ROC) curves and quantified by AUC.

We assessed whether addition of summary measures of the gut microbiome could improve the predictive performance of models based on only fragment-specific information. To this end, we either provided phylum-specific abundances and Shannon diversity indices as fixed, i.e. non-penalized, covariates to the model, or we added these as flexible covariates that were subject to regularized selection, just as individual fragments. In the former case, we omitted the ratio of FAFV to Bacteroidetes from the model, to avoid collinearity among fixed predictors. In the latter case, abundance and diversity measures we subject to distinct penalization through specification of a separate class of co-data. Likewise, we combined all patient characteristics and individual fragment length abundance data obtained by IS-pro, collected either at baseline or at day 5 of treatment, into one predictor. This was done with or without addition of gut microbiome summary measures, added as flexible covariates to the model as described above.

**Table S1. Intestinal microbiota characteristics at baseline (before start of CDI treatment, D0) and on day 5 of CDI treatment (D5).** Median values (and IQR: interquartile ranges) are given separately for patients with and without reCDI. Patient numbers slightly differ per time point since stool samples could not be collected from all patients at all time points.

| **Microbiota characteristic** | **Median (IQR)** | **Median (IQR)** | **P value $** |
| --- | --- | --- | --- |
| **D0** | **No reCDI** (n=142) | **reCDI** (n=45) |  |
| **Microbial abundance** *(x10^5^)* |  |  |  |
| FAFV | 1.81 (1.50; 2.22) | 1.87 (1.50; 2.18) | 0.778 |
| BACT | 1.83 (1.04; 2.27) | 1.79 (1.17; 2.27) | 0.976 |
| PROT | 2.61 (2.19; 3.03) | 2.68 (1.62; 3.09) | 0.861 |
| *C. difficile* | 1.60 (1.31; 1.91) (n=138) | 1.76 (1.53; 1.92) | 0.136 |
| **log Abundance ratio ‡** |  |  |  |
| FAFV:BACT | –0.01 (–0.30; 0.57) | 0.09 (–0.27; 0.33) | 0.778 |
| **Shannon diversity** |  |  |  |
| FAFV | 3.48 (3.20; 3.74) | 3.44 (3.24; 3.67) | 0.916 |
| BACT | 3.11 (2.61; 3.48) | 2.92 (2.63; 3.31) | 0.199 |
| PROT | 3.50 (3.26; 3.65) | 3.47 (3.15; 3.62) | 0.493 |
| **D5** | **No reCDI** (n=144) | **reCDI** (n=48) |  |
| **Microbial abundance** *(x10^5^)* |  |  |  |
| FAFV | 1.84 (1.49; 2.16) | 1.89 (1.63; 2.24) | 0.191 |
| BACT | 0.43 (0.02; 1.35) | 0.24 (0.04; 0.71) | 0.711 |
| PROT | 2.30 (1.03; 2.91) | 2.45 (1.75; 3.03) | 0.224 |
| **log Abundance ratio ‡** |  |  |  |
| FAFV:BACT | 1.56 (0.28; 4.15) | 2.09 (0.99; 3.64) | 0.597 |
| **Shannon diversity** |  |  |  |
| FAFV | 3.15 (2.87; 3.39) | 3.16 (2.86; 3.37) | 0.899 |
| BACT | 2.37 (1.10; 3.03) | 1.93 (1.60; 2.46) | 0.362 |
| PROT | 3.30 (2.81; 3.55) | 3.51 (3.15; 3.68) | 0.012 |
| **Difference between D0 and D5** | **No reCDI** (n=130) | **reCDI** (n=42) |  |
| **Microbial abundance** *(x10^5^)* |  |  |  |
| FAFV | -0.07 (-0.44; 0.49) | 0.02 (-0.31; 0.51) | 0.384 |
| BACT | -0.91 (-1.71; -0.08) | -1.19 (-1.72; -0.34) | 0.222 |
| PROT | -0.30 (-1.20; 0.16) | -0.03 (-0.64; 0.55) | 0.019 |
| **log Abundance ratio ‡** |  |  |  |
| FAFV:BACT | 0.41 (0.04; 1.60) | 0.77 (0.33; 1.40) | 0.282 |
| **Shannon diversity** |  |  |  |
| FAFV | -0.32 (-0.61; -0.07) | -0.25 (-0.66; -0.08) | 0.785 |
| BACT | -0.53 (-1.42; -0.03) | -0.62 (-1.23; -0.17) | 0.877 |
| PROT | -0.16 (-0.76; 0.09) | 0.14 (-0.19; 0.36) | <0.001 |

‡ *Abundance ratio is given after natural log transformation: negative values denote ratios below 1; $ p-values from Mann-Whitney U test without correction for multiple testing*

**Table S2. Clinical factors associated with absolute microbial abundance** **at baseline or day 5 of CDI treatment.** Variables are listed for which at least one dichotomous comparison yielded *p* < 0.05 by the Mann-Whitney U test.

| **Clinical factors associated with abundance at D0** | **Phylum** | **Median (IQR) ^ Abundance** | **P value $** |
| --- | --- | --- | --- |
| Gender (male vs. female) | BACT | 1.87 (1.24; 2.45) vs. 1.58 (0.88; 2.15) | 0.007 |
| Tobacco (no vs. yes) | PROT | 2.68 (2.19; 3.14) vs. 2.43 (1.91; 2.92) | 0.033 |
| IBD (no vs. yes) | FAFV | 1.79 (1.49; 2.15) vs. 2.27 (1.75; 2.43) | 0.008 |
| Immunocompromised (no vs. yes) | FAFV | 1.76 (1.49; 2.06) vs. 1.91 (1.59; 2.25) | 0.046 |
| Stool type (formed, Bristol stool scale 1-4, vs. loose, Bristol stool scale 5-7) | PROT | 2.91 (2.47; 3.53) vs. 2.56 (1.98; 2.97) | 0.012 |
| *Prior use of: ‡* |  |  |  |
| 1^st^ generation cephalosporines (no vs. yes) | BACT | 1.77 (1.02; 2.25) vs. 2.04 (1.73; 2.57) | 0.018 |
| carbapenems (no vs. yes) | BACT | 1.82 (1.12; 2.28) vs. 0.96 (0.20; 1.40) | 0.009 |
| metronidazole (no vs. yes) | FAFV  BACT | 1.79 (1.48; 2.08) vs. 1.93 (1.71; 2.34)  1.83 (1.29; 2.29) vs. 1.15 (0.28; 2.15) | 0.034  0.009 |
| cotrimoxazole (no vs. yes) | FAFV  BACT | 1.77 (1.49; 2.18) vs. 1.99 (1.82; 2.24)  1.82 (1.13; 2.29) vs. 1.20 (0.55; 1.92) | 0.039  0.039 |
| vancomycin/teicoplanine (no vs. yes) | FAFV | 1.79 (1.49; 2.17) vs. 2.17 (1.89; 2.37) | 0.012 |
| **Clinical factors associated with abundance at D5** | **Phylum** | **Median (IQR) ^ Abundance** | **P value $** |
| IBD (no vs. yes) | FAFV  PROT | 1.83 (1.49; 2.14) vs. 1.98 (1.85; 2.31)  2.29 (1.09; 2.90) vs. 2.70 (2.53; 3.57) | 0.043  0.017 |
| Proton pump inhibitor (no vs. yes) | FAFV | 1.77 (1.48; 2.06) vs. 1.93 (1.55; 2.25) | 0.028 |
| Hospitalization (no vs. yes) | PROT | 2.53 (2.04; 3.15) vs. 2.31 (1.06; 2.88) | 0.038 |
| Hypervirulent strain (no vs. yes) | FAFV | 1.84 (1.50; 2.16) vs. 2.11 (2.03; 2.28) | 0.049 |
| *Prior use of: ‡* |  |  |  |
| 3^rd^ generation cephalosporines (no vs. yes) | PROT | 2.44 (1.74; 2.90) vs. 2.03 (0.52; 2.93) | 0.040 |
| metronidazole (no vs. yes) | BACT | 0.46 (0.03; 1.33) vs. 0.04 (0.02; 0.32) | 0.002 |
| fluoroquinolones (no vs. yes) | FAFV  PROT | 1.91 (1.53; 2.26) vs. 1.74 (1.34; 1.90)  2.44 (1.37; 3.06) vs. 1.96 (0.84; 2.61) | 0.006  0.006 |
| selective digestive decontamination (no vs. yes) | BACT | 0.29 (0.03; 1.10) vs. 1.50 (0.72; 2.29) | 0.005 |
| Antibiotic continuation at D5 (none vs. any) | FAFV  PROT | 1.90 (1.63; 2.22) vs. 1.70 (1.34; 2.08)  2.51 (1.35; 3.06) vs. 2.07 (0.75; 2.77) | 0.014  0.019 |
| CDI antibiotic (metronidazole vs. vancomycin) | BACT | 0.06 (0.02; 0.92) vs. 0.52 (0.07; 1.15) | 0.009 |

^ *Median (interquartile range) in reference (first category listed) versus comparator; $ p-values without correction for multiple testing**;* ‡ *Within 3 months from start of CDI treatment*

**Table S3. Clinical factors associated with FAFV:BACT abundance ratio at baseline or day 5 of CDI treatment.** Variables are listed for which at least one dichotomous comparison yielded *p* < 0.05 by the Mann-Whitney U test.

| **Clinical factors associated with FAFV:BACT ratio at D0** | **Median (IQR) ^ log Abundance ratio *** | **P value $** |
| --- | --- | --- |
| Gender (male vs. female) | –0.06 (–0.38; 0.29) vs. 0.09 (–0.20; 0.74) | 0.009 |
| *Prior use of: ‡* |  |  |
| 3^rd^ generation cephalosporines (no vs. yes) | –0.04 (–0.30; 0.33) vs. 0.10 (–0.20; 0.86) | 0.039 |
| carbapenems (no vs. yes) | –0.03 (–0.32; 0.34) vs. 0.50 (–0.04; 1.84) | < 0.001 |
| aminoglycosides (no vs. yes) | –0.02 (–0.31; 0.47) vs. 0.31 (0.07; 0.55) | 0.028 |
| cotrimoxazole (no vs. yes) | –0.02 (–0.32; 0.43) vs. 0.45 (0.03; 1.51) | 0.010 |
| Antibiotic continuation at D5 (none vs. any) | –0.03 (–0.38; 0.32) vs. 0.11 (–0.16; 0.69) | 0.049 |
| **Clinical factors associated with FAFV:BACT ratio at D5** | **Median (IQR) ^ log Abundance ratio *** | **P value $** |
| *Prior use of: ‡* |  |  |
| metronidazole (no vs. yes) | 1.29 (0.28; 3.79) vs. 3.67 (1.56; 4.78) | 0.002 |
| selective digestive decontamination (no vs. yes) | 1.81 (0.56; 4.01) vs. 0.49 (–0.06; 1.09) | 0.014 |
| CDI antibiotic (metronidazole vs. vancomycin) | 3.32 (0.62; 4.60) vs. 1.18 (0.40; 2.58) | 0.007 |

^ *Median (interquartile range) in reference (first category listed) versus comparator;* * *Abundance ratio is given after natural log transformation: negative value denote ratios below 1; $ p-values without correction for multiple testing;* ‡ *Within 3 months from start of CDI treatment*

**Table S4. Clinical factors associated with Shannon diversity at baseline or day 5 of CDI treatment.** Variables are listed for which at least one dichotomous comparison yielded *p* < 0.05 by the Mann-Whitney U test.

| **Clinical factors associated with diversity at D0 †** | **Phylum** | **Median (IQR) ^ Shannon diversity *** | **P value $** |
| --- | --- | --- | --- |
| Age (max 65 years vs. older) | BACT  PROT | 2.91 (2.42; 3.27) vs. 3.18 (2.75; 3.49)  3.43 (3.19; 3.59) vs. 3.53 (3.36; 3.69) | 0.006  0.012 |
| Gender (male vs. female) | BACT | 3.14 (2.77; 3.51) vs. 2.98 (2.17; 3.35) | 0.008 |
| Tobacco (no vs. yes) | PROT | 3.50 (3.31; 3.67) vs. 3.41 (3.05; 3.56) | 0.024 |
| IBD (no vs. yes) | FAFV | 3.45 (3.20; 3.72) vs. 3.65 (3.56; 3.87) | 0.020 |
| Immunocompromised (no vs. yes) | BACT | 3.16 (2.75; 3.48) vs. 2.89 (2.37; 3.33) | 0.014 |
| Hospitalization (no vs. yes) | FAFV | 3.61 (3.45; 3.83) vs. 3.42 (3.20; 3.72) | 0.018 |
| Enteral feeding (no vs. yes) ‡ | FAFV | 3.51 (3.28; 3.75) vs. 3.27 (3.05; 3.63) | 0.005 |
| *Prior use of: ‡* |  |  |  |
| carbapenems (no vs. yes) | PROT | 3.50 (3.26; 3.66) vs. 3.15 (2.60; 3.55) | 0.030 |
| metronidazole (no vs. yes) | BACT  PROT | 3.16 (2.74; 3.45) vs. 2.75 (2.38; 3.10)  3.52 (3.26; 3.68) vs. 3.43 (3.13; 3.58) | 0.009  0.047 |
| aminoglycosides (no vs. yes) | BACT | 3.09 (2.64; 3.44) vs. 2.61 (2.37; 2.82) | 0.017 |
| cotrimoxazole (no vs. yes) | BACT | 3.11 (2.67; 3.48) vs. 2.68 (1.98; 2.95) | 0.001 |
| anaerobe antibiotics (no vs. yes) | FAFV  BACT | 3.54 (3.35; 3.72) vs. 3.40 (3.12; 3.73)  3.24 (2.98; 3.50) vs. 2.90 (2.38; 3.39) | 0.041  0.004 |
| Antibiotic continuation at D5 (none vs. any) † | FAFV | 3.54 (3.28; 3.76) vs. 3.36 (3.11; 3.68) | 0.025 |
| **Clinical factors associated with diversity at D5 †** | **Phylum** | **Median (IQR) ^ Shannon diversity *** | **P value $** |
| IBD (no vs. yes) | PROT | 3.33 (2.83; 3.58) vs. 3.54 (3.33; 3.66) | 0.030 |
| Appendectomy (no vs. yes) | FAFV | 3.15 (2.86; 3.35) vs. 3.27 (3.06; 3.53) | 0.028 |
| Chemotherapy (no vs. yes) | PROT | 3.42 (2.96; 3.61) vs. 3.01 (2.19; 3.28) | 0.009 |
| Stool type (formed vs. loose) | PROT | 3.47 (3.02; 3.68) vs. 3.30 (2.80; 3.55) | 0.029 |
| Hospitalization (no vs. yes) | PROT | 3.52 (3.23; 3.75) vs. 3.33 (2.75; 3.58) | 0.005 |
| *Prior use of: ‡* |  |  |  |
| penicillines (no vs. yes) | FAFV | 3.18 (2.97; 3.46) vs. 3.07 (2.77; 3.30) | 0.016 |
| 3^rd^ generation cephalosporines (no vs. yes) | PROT | 3.44 (3.13; 3.64) vs. 3.15 (2.29; 3.53) | < 0.001 |
| carbapenems (no vs. yes) | PROT | 3.38 (2.92; 3.61) vs. 3.00 (2.29; 3.41) | 0.046 |
| metronidazole (no vs. yes) | BACT | 2.36 (1.60; 3.03) vs. 1.61 (1.09; 2.42) | 0.005 |
| aminoglycosides (no vs. yes) | FAFV | 3.16 (2.92; 3.41) vs. 2.75 (2.60; 2.97) | 0.001 |
| fluoroquinolones (no vs. yes) | FAFV  PROT | 3.16 (2.92; 3.42) vs. 3.05 (2.77; 3.26)  3.43 (3.09; 3.61) vs. 3.02 (2.33; 3.44) | 0.025  0.005 |
| selective digestive decontamination (no vs. yes) | BACT | 2.12 (1.28; 2.93) vs. 2.92 (2.81; 2.95) | 0.034 |
| Antibiotic continuation at D5 (none vs. any) † | FAFV  PROT | 3.24 (2.97; 3.48) vs. 3.06 (2.75 –3.24)  3.44 (3.15; 3.63) vs. 3.12 (2.33 –3.53) | < 0.001  < 0.001 |
| CDI antibiotic (metronidazole vs. vancomycin) | BACT | 1.77 (1.09; 2.69) vs. 2.47 (1.79; 3.03) | 0.001 |

^ *Median (interquartile range) in reference (first category listed) versus comparator; $ p-values without correction for multiple testing;* ‡ *Within 3 months from start of CDI treatment*

**Table S5. Clinical factors associated with specific bacterial species at baseline or day 5 of CDI treatment.** Bacterial species are specified for clinical variables with an effect on microbial abundance or diversity (Tables S2-4) and an AUC ≥0.60. Colors indicate whether the clinical factor is associated with an increase (green) or decrease (red) of a specific bacterial species. First the number and Phylum (FAFV/BACT/PROT) of IS-fragments associated with the clinical factor are listed, and then the bacterial species that were matched to these fragments via the IS-pro species database.

| **Clinical factors** | **Association with bacterial species abundance on D0** | **AUC#** | **Association with bacterial species abundance on D5** | **AUC#** |
| --- | --- | --- | --- | --- |
| Age ≥65 years | 17 FAFV, 15 BACT, 3 PROT;  *Alistipes finegoldii*  *Alistipes putredinis*  *Bacteroides fragilis*  *Klebsiella pneumoniae*  *Prevotella sp.*  *Prevotella buccae*  *Prevotella multisaccharivorax*  *Prevotella veroralis*  *Pseudomonas putida*  *Serratia marcescens*  *Staphylococcus aureus*  *Clostridium sporogenes*  *Clostridium sordellii*  *Clostridium cadaveris*  *Lactobacillus crispatus* | 0.61 | 12 FAFV, 13 BACT;  *Alistipes finegoldii*  *Bacteroides fragilis*  *Bacteroides spp.*  *Bacteroides uniformis*  *Clostridium butyricum*  *Corynebacterium pseudogenitalium*  *Lactobacillus acidophilus/rhamnosus*  *Prevotella spp. PB69*  *Clostridium sordellii*  *Corynebacterium glucuronolyticum*  *Fusobacterium nucleatum*  *Ruminococcus sp.* | 0.63 |
| Hospitalization | 23 FAFV, 2 BACT;  *Bacteroides vulgatus*  *Clostridium butyricum*  *Fusobacterium nucleatum*  *Akkermansia muciniphila*  *Clostridium acetobutylicum*  *Clostridium cadaveris*  *Clostridium sordellii*  *Dialister spp.*  *Eubacterium siraeum*  *Eubacterium ventriosum*  *Faecalibacterium prausnitzii*  *Ruminococcus gnavus* | 0.72 | 11 FAFV, 14 PROT;  *Clostridium butyricum*  *Lactobacillus acidophilus/rhamnosus*  *Staphylococcus carnosus*  *Enterobacteriaceae ISP727*  *Enterococcus faecalis*  *Faecalibacterium prausnitzii*  *Klebsiella oxytoca*  *Proteobacteria ISP 589* | 0.77 |
| IBD | 20 FAFV, 4 BACT, 1 PROT;  *Bacillus spp. ISF192*  *Bacteroides salyersiae*  *Capnocytophaga ochracea*  *Clostridium sporogenes*  *Clostridium sordellii*  *Clostridium perfringens*  *Corynebacterium pseudogenitalium*  *Eubacterium spp. ISF191*  *Lactobacillus crispatus*  *Lactobacillus johnsonii*  *Lactobacillus*  *acidophilus/rhamnosus*  *Ruminococcus flavefaciens*  *Shewanella putrefaciens*  *Veillonella dispar* | 0.64 | 8 FAFV, 3 BACT, 14 PROT;  *Clostridium sordellii*  *Fusobacterium nucleatum*  *Klebsiella pneumoniae*  *Pseudomonas putida*  *Roseomonas cervicalis*  *Sutterella spp.*  *Thioalkalivibrio spp.*  *Clostridium butyricum*  *Clostridium sporogenes*  *Faecalibacterium prausnitzii* | 0.62 |
| Immunocompromised | 6 FAFV, 9 BACT, 10 PROT*;*  *Burkholderia sp.*  *Coprococcus eutactus*  *Corynebacterium pseudogenitalium*  *Sutterella wadsworthensis*  *Alistipes finegoldii*  *Bacteroides ovatus*  *Bacteroides spp.*  *Barnesiella viscericola*  *Escherichia coli*  *Faecalibacterium prausnitzii*  *Parabacteroides distasonis*  *Proteobacteria ISP 589*  *Ruminococcus sp.*  *Thioalkalivibrio spp.* | 0.62 | *-* | *-* |
| Stool type (formed, Bristol stool scale 1-4, vs. loose, Bristol stool scale 5-7) | 23 FAFV, 1 BACT, 1 PROT;  *Akkermansia muciniphila*  *Bacillus spp. ISF192*  *Blautia producta*  *Clostridiales spp.*  *Clostridium bolteae*  *Clostridium cadaveris*  *Eubacterium spp. ISF191*  *Eubacterium siraeum*  *Haemophilus influenzae*  *Lactobacillus johnsonii*  *Megamonas hypermegale*  *Roseburia hominis* | 0.62 | - | - |
| *Prior use of:* ‡ |  |  |  |  |
| 2^nd^ generation cephalosporin | 15 FAFV, 7 BACT, 3 PROT *;*  *Alistipes finegoldii*  *Bacteroides intestinalis*  *Bacteroides thetaiotaomicron*  *Bacteroides uniformis*  *Campylobacter jejuni*  *Clostridium cadaveris*  *Dorea formicigenerans*  *Lactobacillus reuteri*  *Prevotella veroralis*  *Ruminococcus sp.*  *Roseburia hominis*  *Serratia proteamaculans*  *Streptococcus spp. ISF293*  *Streptococcus spp. ISF294* | 0.70 | *-* | *-* |
| 3^rd^ generation cephalosporin | 9 FAFV, 16 PROT*;*  *Bifidobacterium longum*  *Burkholderia sp.*  *Burkholderia multivorans*  *Clostridium cadaveris*  *Proteobacteria ISP545*  *Enterobacteriaceae ISP829*  *Escherichia coli*  *Lactobacillus acidophilus/rhamnosus*  *Lactobacillus johnsonii*  *Ruminococcus bicirculans* | 0.70 | 10 FAFV, 15 PROT;  *Bifidobacterium longum*  *Clostridium butyricum*  *Faecalibacterium prausnitzii*  *Lactobacillus acidophilus/rhamnosus*  *Thioalkalivibrio spp.*  *Clostridium sordellii*  *Enterobacteriaceae ISP829*  *Escherichia coli*  *Eubacterium biforme*  *Fusobacterium nucleatum*  *Klebsiella pneumoniae*  *Proteobacteria ISP 589*  *Sutterella spp.*  *Sutterella wadsworthensis* | 0.72 |
| Carbapenem | 15 FAFV, 6 BACT, 4 PROT*;*  *Bifidobacterium longum*  *Clostridium sporogenes*  *Enterococcus faecalis*  *Ruminococcus sp.*  *Staphylococcus carnosus*  *Alistipes finegoldii*  *Bacteroides ovatus*  *Bacteroides thetaiotaomicron*  *Clostridium sordellii*  *Escherichia coli*  *Faecalibacterium prausnitzii*  *Fusobacterium nucleatum*  *Prevotella spp. PB69* | 0.71 | *-* | *-* |
| Cotrimoxazole | 11 FAFV, 9 BACT, 5 PROT*;*  *Anaerostipes hadrus*  *Bacteroides vulgatus*  *Burkholderia multivorans*  *Corynebacterium pseudogenitalium*  *Eubacterium siraeum*  *Faecalibacterium prausnitzii*  *Serratia marcescens*  *Veillonella dispar*  *Bacteroides caccae*  *Bacteroides fragilis*  *Bacteroides thetaiotaomicron*  *Bacteroides uniformis*  *Blautia producta*  *Clostridium sordellii*  *Dialister invisus*  *Sutterella wadsworthensis* | 0.76 | *-* | *-* |
| Clindamycin | 1 FAFV, 15 BACT, 9 PROT*;*  *Bacteroides thetaiotaomicron*  *Bacteroides spp.*  *Butyricimonas spp.*  *Escherichia coli*  *Parabacteroides merdae*  *Pseudomonas putida*  *Proteobacteria ISP747*  *Proteobacteria ISP904*  *Ruminococcus sp.*  *Thioalkalivibrio spp*  *Bacteroides vulgatus*  *Prevotella copri*  *Parabacteroides distasonis* | 0.70 | *-* | *-* |
| Aminoglycoside | - | - | 25 FAFV;  *Bifidobacterium spp.*  *Clostridiales spp.*  *Clostridium cochlearium*  *Corynebacterium urealyticum*  *Enterococcus faecalis*  *Faecalibacterium prausnitzii*  *Gemella haemolysans*  *Lachnospiraceae incertae sedis*  *Lactobacillus johnsonii*  *Phascolarctobacterium succinatutens*  *Ruminococcus sp.*  *Staphylococcus carnosus*  *Staphylococcus epidermidis*  *Streptococcus equi*  *Corynebacterium glucuronolyticum*  *Eubacterium biforme*  *Faecalibacterium prausnitzii*  *Lactobacillus acidophilus/rhamnosus* | 0.65 |
| Antibiotic against anaerobic bacteria~ | 8 FAFV, 17 BACT*;*  *Faecalibacterium prausnitzii*  *Bacteroides dorei*  *Bacteroides fragilis*  *Bacteroides ovatus*  *Bacteroides vulgatus*  *Eubacterium siraeum*  *Parabacteroides distasonis*  *Prevotella copri*  *Prevotella spp. PB69* | 0.63 | *-* | *-* |
| SDD | 7 FAFV, 8 BACT, 10 PROT*;*  *Alistipes finegoldii*  *Bacteroides fragilis*  *Bacteroides vulgatus*  *Bacteroides uniformis*  *Clostridium butyricum*  *Parabacteroides distasonis*  *Proteus mirabilis*  *Ruminococcus sp.*  *Burkholderia cenocepacia*  *Clostridium sordellii*  *Escherichia coli*  *Faecalibacterium prausnitzii*  *Prevotella sp.*  *Sutterella wadsworthensis*  *Thioalkalivibrio spp.* | 0.70 | *-* | *-* |
| Metronidazole | 11 FAFV, 14 BACT*;*  *Bifidobacterium longum*  *Enterococcus faecalis*  *Fusobacterium nucleatum*  *Alistipes finegoldii*  *Bacteroides fragilis*  *Bacteroides ovatus*  *Bacteroides vulgatus*  *Odoribacter splanchnicus*  *Parabacteroides distasonis* | 0.66 | *-* | *-* |
| Vancomycin/ teicoplanine | 25 PROT*;*  *Acinetobacter haemolyticus*  *Campylobacter jejuni*  *Campylobacter sp.*  *Dialister succinatiphilus*  *Klebsiella pneumoniae* | 0.64 | *-* | *-* |
| Antibiotic continuation between D0 and D5 (none vs. any) | 24 FAFV, 1 BACT;  *Bifidobacterium longum*  *Clostridium sporogenes*  *Corynebacterium urealyticum*  *Staphylococcus aureus*  *Staphylococcus carnosus*  *Bacteroides fragilis*  *Clostridium perfringens*  *Clostridium sordellii*  *Corynebacterium jeikeium*  *Faecalibacterium prausnitzii*  *Lactobacillus acidophilus/rhamnosus*  *Ruminococcus gnavus*  *Staphylococcus lugdunensis* | 0.67 |  |  |
| CDI treatment with vancomycin (vs. metronidazole) | *-* | - | 23 FAFV, 2 BACT;  *Bifidobacterium longum*  *Clostridium sporogenes*  *Corynebacterium jeikeium*  *Dialister invisus*  *Firmicutes ISF257*  *Lactobacillus acidophilus/rhamnosus Enterococcus faecalis*  *Ruminococcus sp*  *Veillonella dispar* | 0.88 |

# *AUC determined by adaptive group-regularized ridge regression with 10-fold cross-validation;* ‡ *Within 3 months before primary CDI diagnosis; ~* *penicillines, 1st generation cephalosporines, carbapenems, clindamycin, macrolides, tetracyclines and/or metronidazole*

**Supplementary text 1. Specification of 72 clinical factors included in the prediction models.**

*Variables collected on D0:*

Center/hospital (of primary CDI diagnosis), age, gender, medical history of appendectomy, IBD, diabetes mellitus, country of birth, length, weight, BMI, being hospitalized on D0, admission duration on D0, HCF/community associated/acquired CDI, type of CDI antibiotic, CDI antibiotic delay (time between CDI diagnosis and start of therapy), prior CDI, hypervirulent strain (ribotype 027 or 078), diarrhea frequency, bloody diarrhea on D-1 or D0, creatinine baseline (average historical creatinine value of the patient, calculated by: average creatinine per year divided by the number of years with creatinine data. Average creatinine per year was calculated by average creatinine per month (values of current hospitalization excluded) divided by the number of months with creatinine data).

Within ten days before D0: antibiotics (yes/no, and if yes: number of antibiotic categories).

Within three months before D0: tobacco use, alcohol consumption, previous admissions, IC admission, abdominal surgery, colonoscopy, enteral feeding, immunocompromised, neutropenia, and use of: antacid, PPI, immunosuppressant, chemotherapy, antibiotics (yes/no, and if yes: number of antibiotic categories) antibiotics against anaerobe bacteria, penicillin, 1st gen cephalosporin, 2nd gen cephalosporin, 3rd gen cephalosporin, 4th gen cephalosporin, carbapenem, macrolide, clindamycin, metronidazole, tetracycline, aminoglycoside, fluoroquinolone, cotrimoxazole, vancomycin/teicoplanine, other antibiotics, SDD (selective digestive decontamination).

*Variables collected on D5:*

Diarrhea on D5 (yes/no), diarrhea frequency on D5, bloody diarrhea on D5, alcohol consumption between D0-D5, IC admission D0-D5, immunocompromised D0-D5, antibiotic continuation D0-D5, antibiotic quantity D0-D5, creatinine difference (=% creatinine increase from baseline, calculated by: current creatinine/baseline creatinine*100%), stool type of stool sample collected on D5 (formed: Bristol stool scale 1-4 vs. loose: Bristol stool scale 5-7), severe CDI (based on: body temperature ≥38ᵒC or leukocytes ≥15,000 cells/mm^3^ or creatinine increase ≥50% from baseline; for calculation of creatinine increase see variables collected on D0)

Lowest measure between D-2 and D2: systolic blood pressure, diastolic blood pressure.

Highest measure between D-2 and D2: heart frequency, temperature, leukocytes, CRP, creatinine.

**Figure S1.** Effect direction/partial effect plots of the twenty most important clinical factors for prediction of reCDI. The higher the partial effect (Y-axis), the higher the chance of reCDI.

**
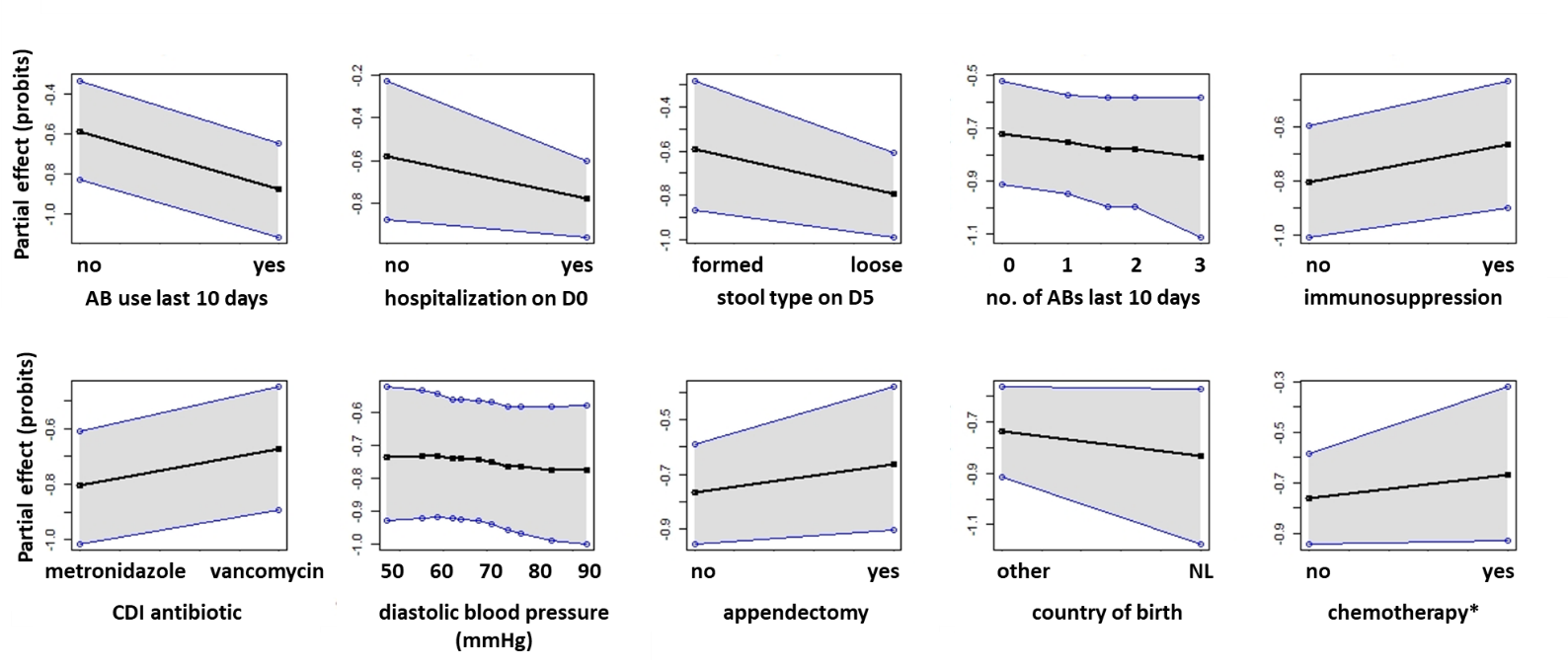
**

**
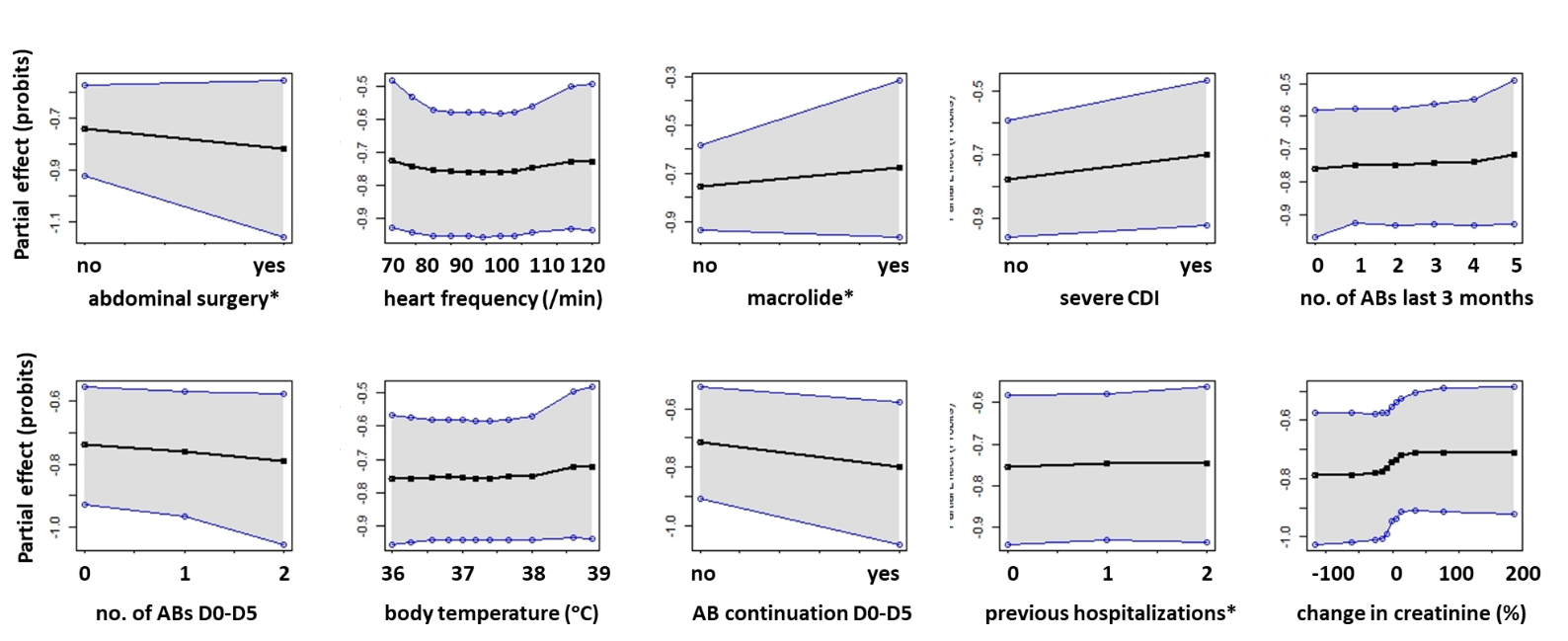
**

**Figure S2.** Effect direction/partial effect plots of the twenty most important microbial factors for prediction of reCDI. The higher the partial effect (Y-axis), the higher the chance of reCDI.

**
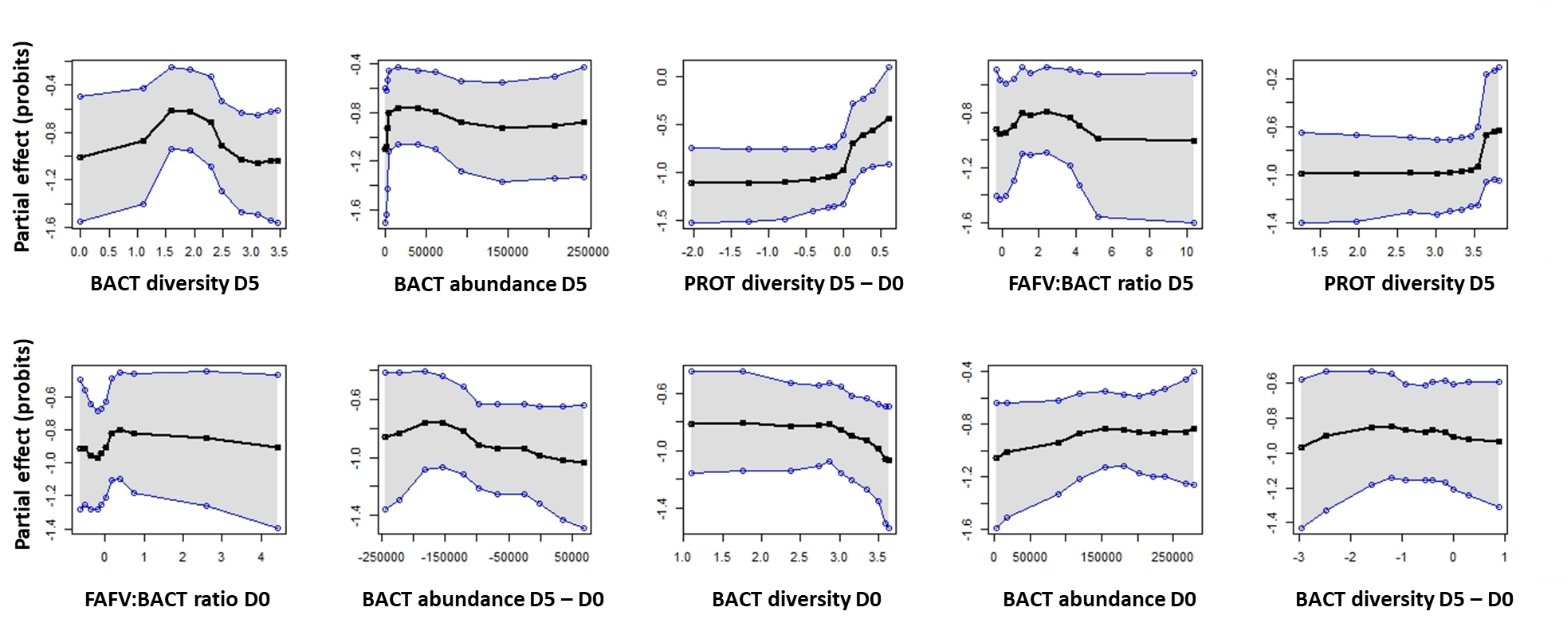
**

**
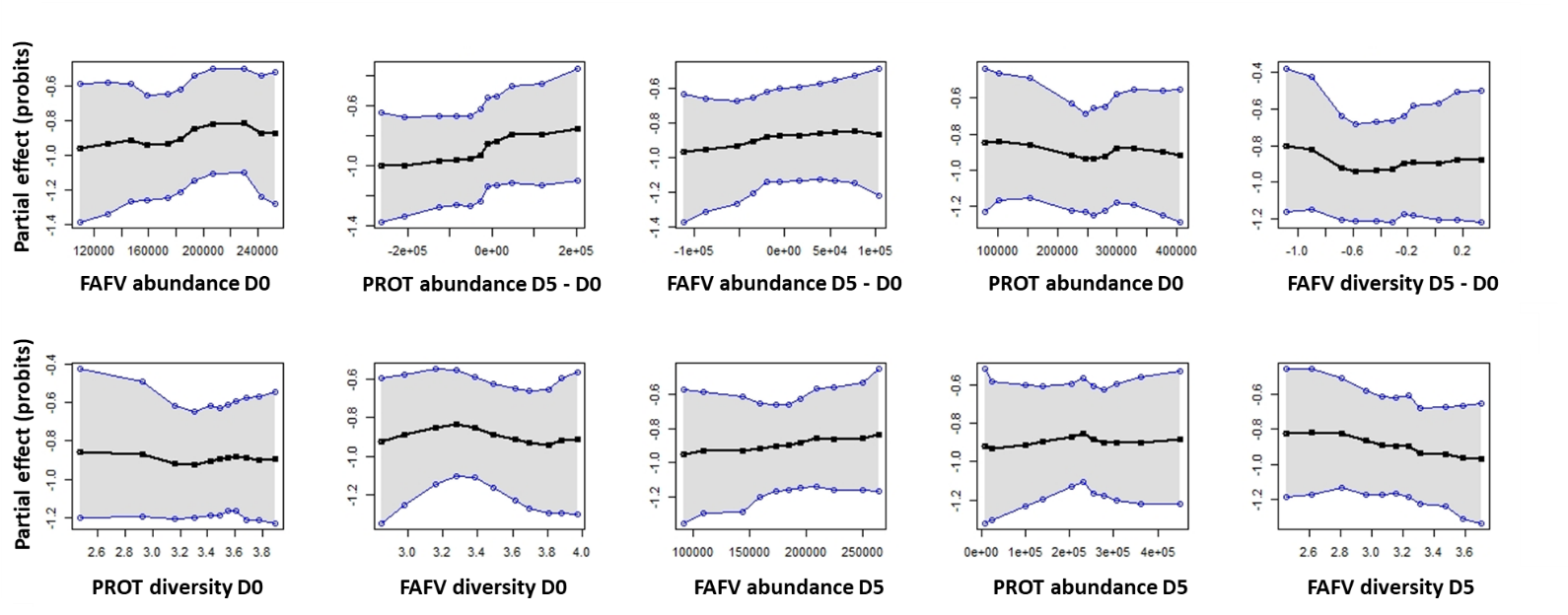
**

**Table S6.** Overview of prediction models not included in the manuscript. Colors indicate which factor increases (orange) or decreases (green) reCDI risk. First the number and Phylum (FAFV/BACT/PROT) of IS-fragments associated with reCDI are listed, and then the bacterial species that were matched to these fragments via the IS-pro species database.

| **Prediction based on:** | **AUC** | **AUC after cross-validation** | **Specification of most distinctive factors^** |
| --- | --- | --- | --- |
| Change in bacterial species between D0-D5 | 0.99 | 0.41 | 10 FAFV, 5 BACT, 10 PROT;  *Clostridium putrefaciens, Eubacterium hallii, Parvimonas micra, Acinetobacter haemolyticus, Burkholderia cenocepacia, Corynebacterium jeikeium, Klebsiella oxytoca, Klebsiella pneumonia, Prevotella sp, Proteobacteria ISP 612, Pseudomonas aeruginosa* |
| Bacterial species on D5 | 0.97 | 0.42 | 5 FAFV, 6 BACT, 14 PROT;  *Burkholderia pseudomallei, Desulfovibrio vulgaris, Eubacterium rectale, Haemophilus parainfluenzae, Klebsiella oxytoca, Proteobacteria ISP798, Ruminococcus flavefaciens, Stenotrophomonas maltophilia, Bacteroides spp., Bacteroides vulgatus, Barnesiella viscericola, Prevotella copri* |
| Clinical factors and bacterial species on D5 | 0.96 | 0.44 | 3 FAFV, 5 BACT, 16 PROT, **no. of antibiotics last 10 days;**  *Burkholderia pseudomallei, Desulfovibrio vulgaris, Eubacterium rectale, Haemophilus parainfluenzae, Klebsiella oxytoca, Neisseria meningitides, Proteobacteria ISP798, Ruminococcus flavefaciens, Stenotrophomonas maltophilia, Bacteroides spp., Bacteroides vulgatus, Barnesiella viscericola, Prevotella copri* |
| Preselection of clinical factors‡ and flexible selection of bacterial species on D5 | 0.82 | 0.54 | 7 FAFV, 3 BACT, 1 PROT; **hospitalized, stool type, antibiotics last 10 days, no. of antibiotics last 10 days;**  *Bifidobacterium longum, Corynebacterium ulcerans, Corynebacterium jeikeium, Ruminococcus flavefaciens, Bacteroides vulgatus, Bacteroides spp., Prevotella spp. PB69, Ruminococcus sp.* |
| Bacterial species and flexible abundance/diversity measures at D0 $ | 1. | 0.48 | 13 FAFV, 9 BACT, 3 PROT;  *Bacteroides fragilis, Bacteroides thetaiotaomicron, Clostridium perfringens, Coprococcus eutactus, Desulfovibrio vulgaris, Dialister hominis, Dialister spp., Micrococcus luteus, Parabacteroides merdae, Ruminococcus gnavus, Prevotella fusca, Streptococcus equi* |
| Bacterial species and flexible abundance/diversity measures at D5 $ | 0.97 | 0.40 | FAFV, 3 BACT, 11 PROT**; FAFV abundance, BACT abundance, PROT abundance;**  *Burkholderia pseudomallei, Corynebacterium ulcerans, Eubacterium spp. ISF191, Eubacterium rectale, Haemophilus parainfluenzae, Klebsiella oxytoca, Ruminococcus flavefaciens, Shewanella putrefaciens,*  *Stenotrophomonas maltophilia, Bacteroides spp., Bacteroides vulgatus* |
| Bacterial species and fixed abundance/diversity measures at D0 $ | 0.97 | 0.50 | 15 FAFV, 7 BACT, 3 PROT;  *Akkermansia muciniphila, Bacteroides thetaiotaomicron, Clostridium butyricum, Clostridium perfringens, Coprococcus eutactus, Desulfovibrio vulgaris, Dialister spp., Fusobacterium nucleatum, Micrococcus luteus, Parabacteroides merdae, Ruminococcus gnavus, Faecalibacterium prausnitzii, Prevotella fusca, Streptococcus equi* |
| Bacterial species and fixed abundance/diversity measures at D5 $ | 0.66 | 0.56 | 20 FAFV, 3 BACT, 2 PROT;  *Corynebacterium ulcerans, Eubacterium spp. ISF191, Eubacterium rectale, Fusobacterium nucleatum, Kytococcus sedentarius, Ruminococcus flavefaciens, Atopobium rimae, Ruminococcus sp.* |
| Clinical factors, bacterial species and flexible abundance/diversity on D0 | 1. | 0.51 | 12 FAFV, 5 BACT, 2 PROT, **CDI antibiotic=vancomycin, hospitalized, immunocompromised, stool type, antibiotics last 10 days, no. of antibiotics last 10 days;**  *Bacteroides fragilis, Bacteroides thetaiotaomicron, Clostridium perfringens, Coprococcus eutactus, Desulfovibrio vulgaris, Dialister hominis, Dialister spp., Micrococcus luteus, Parabacteroides merdae, Ruminococcus gnavus, Streptococcus equi* |
| Clinical factors, bacterial species and flexible abundance/diversity on D5 | 0.96 | 0.40 | 7 FAFV, 2 BACT, 11 PROT, **PROT abundance, hospitalized, stool type, antibiotics last 10 days, no. of antibiotics last 10 days;**  *Burkholderia pseudomallei, Corynebacterium ulcerans, Eubacterium rectale, Haemophilus parainfluenzae, Klebsiella oxytoca, Ruminococcus flavefaciens, Shewanella putrefaciens, Stenotrophomonas maltophilia, Bacteroides spp., Bacteroides vulgatus* |
| Preselection of clinical factors‡, flexible selection of bacterial species and fixed$ inclusion of microbial abundance/diversity on D5 | 0.77 | 0.63 | 5 FAFV, 2 BACT, 8 PROT; **hospitalized, stool type, antibiotics last 10 days, no. of antibiotics last 10 days;**  *Bacteroides vulgatus, Burkholderia pseudomallei, Corynebacterium ulcerans (2x), Haemophilus parainfluenzae, Klebsiella oxytoca (2x), Proteobacteria ISP545, Ruminococcus flavefaciens,* |

*‡ Pre-selection of patient characteristics by Bayesian additive regression trees (BART) within cross-validation; $ Fixed summary measures are not penalized, whereas flexible summary measures are penalized*

**Table S7.** Assessment of the optimum number of clinical factors in combination with bacterial species (AGRR prediction model).

| **Number of predictors** | **AUC** |
| --- | --- |
| 0 | 0,46 |
| 1 | 0,55 |
| 2 | 0,61 |
| 3 | 0,67 |
| 4 | 0,65 |
| 5 | 0,65 |
| 6 | 0,63 |
| 7 | 0,60 |
| 10 | 0,54 |
| 20 | 0,52 |
| 72 | 0,49 |

**Figure S3.** Assessment of the optimum number of clinical factors in combination with bacterial species (AGRR prediction model). Y-axis: Area under the curve (auc), X-axis: number of clinical predictors included in the model. **
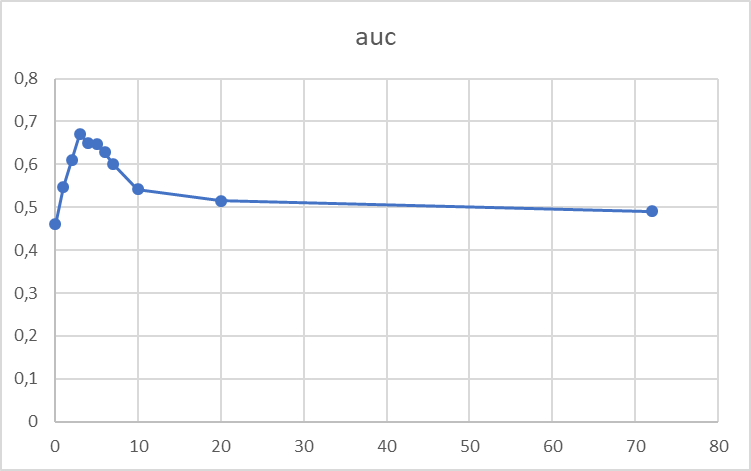
**

**Supplementary text 2. Sensitivity analyses.**

We performed several sensitivity analyses: stratified by CDI antibiotic (metronidazole or vancomycin), excluding IBD patients, excluding primary CDI-treatment non-responders, and lastly, excluding fidaxomicin users and using a shortened list of clinical factors by combining overlapping factors (such as chemotherapy and immunocompromised), but this did not lead to significantly better prediction models. Below the results of the sensitivity analyses, each displayed in an ‘adjusted Table 3’ of the manuscript. Green percentages indicate a higher accuracy than the original model (base case), red percentages indicate a lower accuracy than the original model.

**SENSITIVITY ANALYSIS I. Metronidazole users only.**

**Adjusted Table 3.** Prediction of reCDI by clinical factors and/or microbial abundance/diversity at baseline or D5 of CDI treatment **for metronidazole users only (n=115, 24 with reCDI)**. Predictions are obtained by Bayesian additive regression trees (BART) with choice of the hyperparameters based on cross-validation.

|  | **Before cross-validation** | | **After cross-validation** | |
| --- | --- | --- | --- | --- |
| **Prediction based on:** | **Sensitivity** | **Specificity** | **Sensitivity** | **Specificity** |
| Clinical factors* | **96%** | **69%** | **50%** | **51%** |
| Microbial abundance/diversity † | **100%** | **79%** | **59%** | **61%** |
| Clinical factors and microbial abundancy/diversity ‡ | **100%** | **88%** | **47%** | **61%** |

Green: higher than base case; red: lower than base case

* AB use last 10 days still appears as most important risk factor** for reCDI: associated with decreased risk.

Appendectomy, Immunosuppressants, Immunocompromised remain all associated with increased reCDI risk.

Previous use vancomycin/teicoplanine is now also associated with increased risk: OR = 2.83 (univariate). Hospitalization and stool type are no longer important. [**NB neutropenia was the most important risk factor for reCDI but only has 3 cases]

† BACT D5 abundance and diversity still appear as most important predictors for reCDI: same nonlinear shape.

PROT diversity D5 – D0, FAFV:BACT ratio D5 remain next important predictors: also same nonlinear shape.

BACT diversity D0 and FAFV diversity D5 now more important than PROT diversity D5.

‡ Clinical factors now appear as more important predictors for reCDI than microbiota summary measures.

Neutropenia still no.1, Immunosuppressants, Immunocompromised, Tobacco associated with increased risk.

Previous use vancomycin/teicoplanine selected before AB use last 10 days or microbiota summary measures.

**SENSITIVITY ANALYSIS II. Vancomycin users only.**

**Adjusted Table 3.** Prediction of reCDI by clinical factors and/or microbial abundance/diversity at baseline or D5 of CDI treatment **for vancomycin users only (n=89, 27 with reCDI)**. Predictions are obtained by Bayesian additive regression trees (BART) with choice of the hyperparameters based on cross-validation.

|  | **Before cross-validation** | | **After cross-validation** | |
| --- | --- | --- | --- | --- |
| **Prediction based on:** | **Sensitivity** | **Specificity** | **Sensitivity** | **Specificity** |
| Clinical factors* | **89%** | **73%** | **63%** | **58%** |
| Microbial abundance/diversity † | **100%** | **76%** | **71%** | **65%** |
| Clinical factors and microbial abundancy/diversity ‡ | **100%** | **80%** | **63%** | **61%** |

Green: higher than base case; red: lower than base case

* Hospitalization becomes most important risk factor for reCDI: OR = 0.09; 95% CI 0.01–0.39 (univariate).

No. of antibiotics last 10 days is 2^nd^ most important risk factor, stool type is 3^rd^ most important.

AB use last 10 days only appears at no. 4, before AB continuation D0 – D5.

† PROT diversity D5 – D0 and PROT diversity D5 become most important predictors: still same nonlinear shape.

BACT D5 abundance and diversity now appear as next most important predictors shape FAFV:BACT ratio D5.

FAFV:BACT ratio D5 becomes less important.

‡ Clinical factors now appear as more important predictors for reCDI than microbiota summary measures. Hospitalized still no.1, before no. of antibiotics last 10 days and stool type.

PROT diversity D5 – D0 and PROT diversity D5 are next, still top 5.

**SENSITIVITY ANALYSIS III. Excluding IBD patients.**

**Adjusted Table 3.** Prediction of reCDI by clinical factors and/or microbial abundance/diversity at baseline or D5 of CDI treatment **for non-IBD patients only (n=192, 46 with reCDI)**. Predictions are obtained by Bayesian additive regression trees (BART) with choice of the hyperparameters based on cross-validation.

|  | **Before cross-validation** | | **After cross-validation** | |
| --- | --- | --- | --- | --- |
| **Prediction based on:** | **Sensitivity** | **Specificity** | **Sensitivity** | **Specificity** |
| Clinical factors* | **89%** | **77%** | **48%** | **60%** |
| Microbial abundance/diversity † | **97%** | 78% | **58%** | **68%** |
| Clinical factors and microbial abundancy/diversity ‡ | **94%** | **79%** | **47%** | **63%** |

Green: higher than base case; red: lower than base case

* Stool type (loose) become most important risk factor for reCDI: OR = 0.43; 95% CI 0.20–0.93 (univariate).

Hospitalized and AB use last 10 days appear as next most important risk factors: both p<0.05 univariate.

Abdominal surgery no. 5: associated with decreased risk for reCDI.

† More or less the same as in original model.

‡ Microbiota summary measures are again more important predictors for reCDI than clinical factors.

No. of antibiotics last 10 days is the most important clinical factor but only comes in at 6^th^ place.

**SENSITIVITY ANALYSIS IV. Excluding primary CDI-treatment non-responders.**

**Adjusted Table 3.** Prediction of reCDI by clinical factors and/or microbial abundance/diversity at baseline or D5 of CDI treatment **for initial CDI-treatment responders only (n=198, 41 with reCDI)**. Predictions are obtained by Bayesian additive regression trees (BART) with choice of the hyperparameters based on cross-validation.

|  | **Before cross-validation** | | **After cross-validation** | |
| --- | --- | --- | --- | --- |
| **Prediction based on:** | **Sensitivity** | **Specificity** | **Sensitivity** | **Specificity** |
| Clinical factors* | **93%** | **75%** | **63%** | **60%** |
| Microbial abundance/diversity † | **94%** | **75%** | **56%** | **64%** |
| Clinical factors and microbial abundancy/diversity ‡ | **97%** | **78%** | **59%** | **65%** |

Green: higher than base case; red: lower than base case

* CDI antibiotic become 2^nd^ most important risk factor: OR (vancomycin) = 1.95; 95% CI 0.97–3.96 (univariate).

Hospitalized and stool type remain in top-3, before AB use last 10 days and no. of antibiotics: all p<0.05.

† PROT diversity D5 – D0 becomes most important predictor, otherwise top-5 predictors are all the same.

‡ Microbiota summary measures remain more important predictors for reCDI, but somewhat more mixing.

PROT diversity D5 – D0 still most important predictor, PROT diversity D5 and BACT diversity D5 in top-5.

CDI antibiotic now comes in at no. 2 and no. of antibiotics last 10 days at no.3.

**SENSITIVITY ANALYSIS V. Excluding fidaxomicin users and using a shortened list of clinical factors (by combining overlapping factors such as *chemotherapy* and *immunocompromised*)**

**Adjusted Table 3.** Prediction of reCDI by clinical factors and/or microbial abundance/diversity at baseline or D5 of CDI treatment **for vanco/metro users only (n=204, 51 with reCDI) and a shortened list of clinical factors (p=50)**. Predictions are obtained by Bayesian additive regression trees (BART) with choice of the hyperparameters based on cross-validation.

|  | **Before cross-validation** | | **After cross-validation** | |
| --- | --- | --- | --- | --- |
| **Prediction based on:** | **Sensitivity** | **Specificity** | **Sensitivity** | **Specificity** |
| Clinical factors* | **82%** | 71% | **59%** | **54%** |
| Microbial abundance/diversity † | 95% | **76%** | **63%** | 62% |
| Clinical factors and microbial abundancy/diversity ‡ | **98%** | **78%** | **61%** | **56%** |

Green: higher than base case; red: lower than base case

* AB use last 10 days no longer present, AB use last 3 months (incl. 10 days) no longer relevant.

AB quantity and duration D0 – D5 increase in importance: both reduce risk of reCDI.

† More or less the same as in original model.

‡ Microbiota summary measures remain more important predictors for reCDI, perhaps even more so.

BACT diversity and abundance D5 plus PROT diversity D5 – D0 are still top-3.

CDI antibiotic most important clinical factor at no. 4.

**Supplementary text 3. Interactions between different bacterial phyla.**Since Bacteroidetes abundance and Proteobacteria diversity were the two most important predictors of reCDI in BART, this raised curiosity about the co-occurrence between different bacterial phyla within regression trees. As shown in **Figure S4**, the most frequently co-occurring microbiota features were Bacteroidetes abundance and Bacteroidetes diversity on day 5. The combinations of the most important predictors of reCDI across phyla (Bacteroidetes abundance and diversity on day 5 with the difference in Proteobacteria diversity between day 5 and day 0) were not among the 10 most frequently co-occurring variables within regression trees, indicating that these factors were largely independent predictors of reCDI.

**Figure S4. Most important interactions between different bacterial phyla.** Variable importance plot. Values on X-axis: relative measure for the co-occurrence of both predictors in one regression tree.

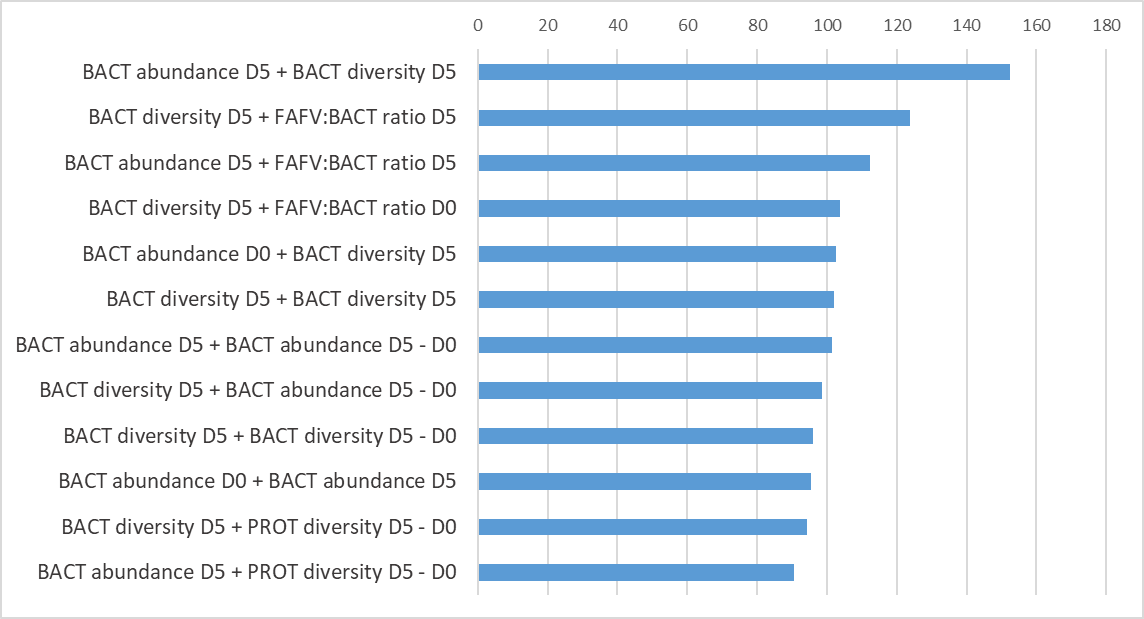

Supplement: Supplementary file 1 — Supplementary Material 1 [file 12879_2024_9506_MOESM1_ESM.docx]
